# Knowledge and attitudes of German and Swiss community pharmacists towards biologicals and biosimilars – a prospective survey before and after the COVID-19 pandemic

**DOI:** 10.1186/s12913-023-10475-x

**Published:** 2023-12-18

**Authors:** Kirstin Messner, Christiane Eickhoff, Martin Schulz, Samuel S. Allemann, Isabelle Arnet

**Affiliations:** 1 Pharmaceutical Care Research Group, University of Basel, Basel, Switzerland; 2 Department of Medicine, ABDA – Federal Union of German Associations of Pharmacists, Berlin, Germany; 3 Institute of Pharmacy, Freie Universität Berlin, Berlin, Germany

**Keywords:** Biological, Biosimilars, Survey, Community pharmacy, Knowledge, Attitudes, Covid-19 pandemic

## Abstract

**Background:**

Knowledge, attitudes and substitution laws of biosimilars are not consistent across countries. Biosimilar acceptance among patients and healthcare professionals may be suffering from gaps in knowledge and understanding about biosimilars and their regulatory approval process. Pharmacists' roles and responsibilities changed considerably during the COVID-19 pandemic. Thus, they might have gained new skills and self-confidence in counseling and substitution of biosimilars.

**Aims:**

To examine and compare the knowledge, perceptions and information needs of German and Swiss pharmacists regarding original biologicals and biosimilars in 2020 and 2022.

**Methods:**

We conducted an online survey among Swiss and German community pharmacies in February 2020 (before) and August 2022 (after the COVID-19 pandemic). Descriptive statistics were calculated and the Chi-Square test was used for comparisons among categorical variables.

**Results:**

A total of 764 pharmacists took part in the survey (390 in 2020 and 374 in 2022) with comparable demographics**.** The frequency of dispensing biologicals remained similar between German and Swiss pharmacists in 2020 and 2022, but the Swiss dispensation of biosimilars increased significantly in 2022 compared to 2020. Concerning the understanding of the term biosimilars, knowledge remained moderate in both countries in both years. Participants were equally familiar with the term and most felt sufficiently informed. In both countries, substitution with a biosimilar showed the least confidence of all attitudes. A third of the participants indicated correct substitution rules in their country. In both years, around 85% of the participants were highly interested in additional training on this topic.

**Discussion/Conclusion:**

The results indicate that similarities and differences between Germany and Switzerland regarding knowledge and attitudes towards biologicals and biosimilars remained unchanged before and after the COVID-19 pandemic. An influence of the pandemic is unlikely. There is still a clear lack of knowledge among community pharmacists on biosimilars, especially regarding the substitution rules. Due to a rising market with many benefits but also big challenges to overcome, the topic of biosimilars should receive more attention in the future. This requires additional training for pharmacists.

**Supplementary Information:**

The online version contains supplementary material available at 10.1186/s12913-023-10475-x.

## Key points

Overall, the similarities and differences between both countries regarding knowledge and attitudes towards biologicals and biosimilars remained unchanged before and after the COVID-19 pandemic. There is still a clear lack of knowledge among community pharmacists on biosimilars, especially regarding the substitution rules. This shows the need for additional training for pharmacists on this topic.

**Supplementary Information:**

The online version contains supplementary material available at 10.1186/s12913-023-10475-x.

## Introduction

A biological pharmaceutical product contains active substances from a biological source, such as living cells or organisms, which are naturally variable. This variability must fall within an acceptable range to ensure consistent safety and efficacy [1]. Biosimilars are biologicals that are developed with the intention to act as alternatives to reference biological products with the same functionality. A biosimilar is highly similar but not identical in its molecular structure due to differences in the complex biotechnological manufacturing process. However, structural differences that may exist of biosimilars compared to their reference product are not clinically relevant [1–4]. Nevertheless, a biosimilar it is not regarded as a generic of a biological medicine. Therefore, biosimilars undergo a different regulatory approval and control process that focuses on analytical and functional comparability in place of extensive clinical study data. In contrast to generics, for which only quality and bioequivalence need to be demonstrated, biosimilars need to demonstrate comparable pharmacokinetics and immunogenicity, with comparable efficacy studies only conducted if there is residual uncertainty [1]. As a result of lower development costs (biologicals: > 800 Mio US-Dollar, biosimilars: 75–250 Mio US-Dollar [5]), biosimilar market uptake can offer advantages to healthcare systems, as it could lead to cost savings and it is expected to improve patient access to costly biological therapies [1, 6].

By the end of 2020, 61 biosimilars in Europe [7] and 29 in the USA [8] had received marketing authorization. Despite the fact that biosimilars are highly comparable and reduce costs, the majority had not been able to reach the anticipated initial market uptake [9]. However, there has been an increase in biosimilar uptake in many countries in recent years. By the end of 2022, biosimilar uptake has exceeded 80% for many molecules for which biosimilars are available [10]. As biosimilars differ from generic medicines, healthcare professionals should be informed of considerations relating to their prescribing practices, switching and interchangeability. However, regulatory frameworks for substituting biosimilars vary largely between countries, and evolve rapidly. Substitution is the dispense of generic medicines instead of the prescribed reference product without the consent or knowledge of the prescribing physician [11]. In Europe, interchangeability refers to the possibility of exchanging one medicine for another medicine that is expected to have the same clinical effect [12]. With a statement in September 2022, the EMA considers that once a biosimilar is approved in the EU it is interchangeable, which means the biosimilar can be used instead of its reference product (or vice versa) or one biosimilar can be replaced with another biosimilar of the same reference product [12]. However, the decision on how to implement interchangeability for example in form of switching or substitution is managed at country level [12]. In Germany, the term bioidentical is used for an interchangeable that only differs in the dosage form and/or packaging. Bioidenticals are identical biosimilars produced on the same production line and sold under different trade names.

Substitution policies at pharmacy-level rest with the EU member states [6, 13]. In Switzerland, substitution for biologicals is not permitted for pharmacists [14]. In Germany, substitution of bioidenticals is permitted since April 2020 [15]. A change in legislation is planned in Germany and Norway regarding permission for substitution of biosimilars [16]. In Germany, the plan for automatic substitution is met with great resistance. Selective substitution only for biosimilars used by the physician may be an alternative [17]. In the United States, the term ‘interchangeable biosimilar’ is a legal definition in legislation for which FDA has developed a guidance. To obtain an interchangeability designation, a manufacturer needs to provide the Food and Drug Administration (FDA) with data or rationale that there is no increased safety risk when switching back and forth between reference product and biosimilar [13, 18]. If a biosimilar has the interchangeability designation, pharmacists can substitute with a biosimilar without involvement of the prescriber.

Reasons for this market uptake below expectations might be the many challenges the biosimilar market has to face such as the reluctance of patients to change medications, medication adherence problems, misinformation about biosimilars, or lack of financial incentives when the biosimilars and reference products are insured in the same manner [19–21]. Furthermore, one of the biggest challenges and current barriers for the use of biosimilars is the overall low acceptance among healthcare providers. Biosimilar acceptance by healthcare professionals and also by patients may be suffering from gaps in knowledge and understanding about biosimilars and their regulatory approval process [22]. There is also a widespread uncertainty about the interchangeability and substitution of biosimilars among pharmacists as observed in a case study with Belgian stakeholders in 2014 [23].

A systematic review in 2020 highlighted that in the past, the research focus in Europe was primarily on the perspectives of the physicians and patients and therefore, their views were predominantly surveyed and analyzed [24] . In contrast, little is known on the knowledge, attitudes and perception of biologicals and biosimilars among pharmacists, even though they are likely to play a more important role in this field in the near future. A French survey with pharmacists in 2017 found that more than half of the pharmacists indicated they had "little knowledge" about biosimilars, community pharmacists being less familiar with biosimilars than hospital pharmacists [25]. A survey with physicians and pharmacists conducted in Ireland in 2017 showed that the majority of pharmacists claimed to be either very familiar or familiar with the term biosimilar, whereas many GPs were unable to define the term or had never heard of it before [22]. A Polish study highlights the concerns expressed by pharmacists, especially regarding immunogenicity and pharmacokinetic properties, and that biosimilars were not identical to the reference product. According to the participants in this study, substitution by pharmacists is not appropriate [26].

These studies show that knowledge, attitudes and substitution laws of biosimilars are not consistent across countries. To gain more insight into these differences, a consortium of nine countries (Australia, Belgium, Denmark, Finland, Germany, Switzerland, Thailand, United Kingdom and the USA) was founded in 2018 to examine the knowledge, perceptions and information needs of pharmacists regarding biologicals and biosimilars. The consortium developed a questionnaire focusing on three topics: (1) how well are pharmacists informed about biologicals, (2) how well are pharmacists informed about the substitution of biologicals, and (3) which perceptions do pharmacists have towards biosimilars [27].

The COVID-19 pandemic led to a disruption of healthcare services and global vaccination programs were launched. Healthcare providers such as pharmacists were invited to play a paramount role in vaccinating and informing the general public, or managing drug shortages e.g., by substitution, among others [28]. Suddenly, pharmacists needed to expand their knowledge regarding vaccines development [29] and in analogy probably also genetic engineering and biotechnological manufacturing processes. They also needed new skills in counseling on biopharmaceutical medicines because of hesitancies and misconceptions against the new vaccines. Therefore, it is conceivable that healthcare providers gained broader knowledge about biotechnological manufacturing processes in general and potential risks and benefits of immunoactive substances such as vaccines, but also biologicals and biosimilars.

We aimed to examine and compare the knowledge, perceptions and information needs between German and Swiss pharmacists regarding original biologicals and biosimilars in 2020 and 2022. The survey results from all countries represented in the consortium are published separately in a commentary [27]. In the following, we will present and discuss the results of the survey in 2020 and its repetition after the COVID-19 pandemic in 2022 in Germany and Switzerland in more detail.

## Methods

An English-language questionnaire was developed by the consortium [27]. It consisted of 17 items on the following topics (see supplementary material): characteristics of the participants (6 items), definition of biosimilars (1 item), frequency of dispensing biologicals and biosimilars (2 items), attitudes towards biosimilars (1 item), substitution and interchangeability of biosimilars (4 items), information sources on biosimilars (3 items). One item on the impact of the COVID-19 pandemic was added in 2022. The answers were collected as yes/no/don't know or level of agreement and frequency on Likert scales. It was translated into German according to standard forward and back translation procedure for use in Germany and German speaking individuals in Switzerland. The online version of the survey was created in REDCap™, a secure web application, and was used for collecting and managing the study data for this survey.

Dissemination of the survey links in Switzerland occurred in February 2020 and June 2022, and in Germany in May 2020 and June 2022. An invitation to the survey was sent out per email in Switzerland to cantonal pharmacists, cantonal pharmacists' associations, Swiss young pharmacist group and Swiss associations of public health and hospital pharmacists. Recipients were asked to forward the email containing the survey link to their members. In Germany, the survey was distributed to approximately 3,000 participants of a mailing list on pharmaceutical care. In 2020, the survey link was valid during 4 weeks (Switzerland) respective 6 weeks (Germany) and one reminder was sent after 17 days (Switzerland) respective 28 days (Germany) in 2020. In 2022, participants had 3 weeks to answer the survey and no reminder was sent since response rate was sufficient within this timeframe.

### Statistical analysis

The minimum sample size per country was set at 100 based on recommendations to allow valid statistical analysis when conducting multivariable analysis [30, 31]. As Switzerland has different language regions, the English and the German versions of the survey were offered to the participants. For the analysis, only completed surveys from community pharmacists were considered. To simplify analysis, time indicating Likert-scales were grouped to build two categories: “ ≥ 2 times a week” (including once or multiple times a day; 2 to 6 times a week) and “ ≤ 1 time a week” (including once a week; less than once a week; never). To minimize the participants’ tendency towards a neutral middle position [32], agreement indicating Likert-scales were given with a 5-point Likert scale. Answers were merged to build three categories—“Agree” (including strongly agree; agree), “Neither agree nor disagree”, and “Disagree” (including disagree; strongly disagree). Six answer options were available for the definition of biosimilars, of which only one option (“a biosimilar is a similar copy of a biological”) was correct. Descriptive statistics were calculated and the Chi-Square test was used for comparisons among categorical variables. To assess the degree to which our results reflect knowledge and attitudes from the overall population, we calculated the margin of error for our sample size with a 95% confidence interval (MOE_95_) and the Z-value 1.96. Because we performed multiple testing, we applied Bonferroni correction to control for type I error for items belonging to the same family. *P*-values considered to indicate statistical significance are < 0.01 (for the item family “frequency of dispensing”); < 0.008 (for the item family “attitudes”); < 0.004 (for the item families “conditions for using” and “training”), and < 0.005 (for the item families “knowledge on substitution rules” and “sources of information”).

## Results

A total of 764 individuals took part in both surveys, with a similar number of responses received in both years (390 individuals in 2020 and 374 individuals in 2022). Two third of the participants were from Switzerland (Table 1). Overall, there were no statistically significant differences in participants’ characteristics between both years (Table 1). A total of 322 (42.1%) completed surveys from community pharmacists were analyzed (2020: 146 and 2022: 176; MOE_95_ ± 6%; Table 1).

**Table 1:** Characteristics of the 764 participants in both surveys (2020 and 2022) in Germany and Switzerland. Percentages of individuals participating refer to the total N participants, all other percentages refer to the n-number of the respective country (DE = Germany; CH = Switzerland)

	**Country**	**2020 (*N* = 390)**	**2022 (*N* = 374)**	***P*-value**
Individuals participating (n [%])	CH	256 [65.6%]	233 [62.3%]	ns
	DE	134 [34.4%]	141 [37.7%]	
Survey completed (n [%])	CH	128 [50%]	154 [66%]	ns
	DE	92 [69%]	82 [58%]	
Community pharmacists (n [%])	CH	62 [48%]	101 [66%]	< 0.05
	DE	84 [91%]	75 [91%]	
Age (mean [range])	CH	43.3 years [26–80]	42.2 years [24–67]	ns
	DE	50.6 years [25–72]	49.6 years [29–72]	
Gender (n [%])	CH	43 female [69.4%]	73 female [69.4%]	ns
	DE	53 female [63.1%]	43 female [57.3%]	
Years of working experience (mean)	CH	17.0 years	16.4 years	ns
	GE	24.9 years	23.1 years	
Language distribution, only in Switzerland (%)	CH	English 38.7% German 61.3%	English 10.9% German 89.1%	< 0.05

### Frequency of dispensing biologicals and biosimilars

**Fig. 1:**
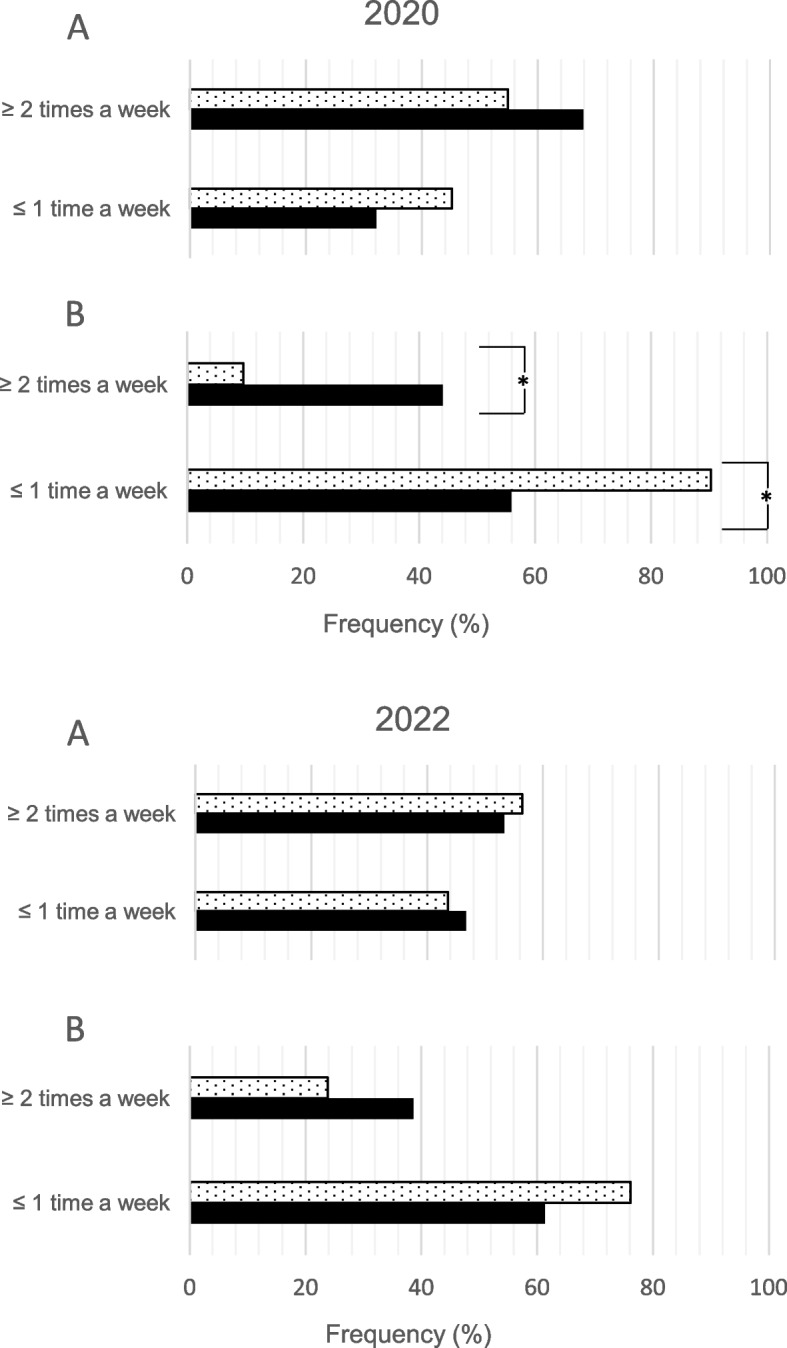
Dispensation of biologicals (panel A) and biosimilars (panel B) by community pharmacists in 2020 and 2022 in Switzerland (dotted bars) and Germany (solid bars) according to the frequencies ≥ 2 times a week and ≤ 1 time a week; statistical significance is marked with an asterisk

In 2020 and 2022, the frequency of dispensing biologicals did not differ significantly between German (DE) and Swiss (CH) pharmacists (2020: ≥ 2 times a week: DE: 67.9% vs CH: 54.8%; ≤ 1 time a week: DE: 32.1% vs CH: 45.2%, ns; 2022: ≥ 2 times a week: DE: 53.3% vs CH: 56.4%; ≤ 1 time a week: DE: 46.7% vs CH: 43.6%, ns; Fig. 1, panel A). Concerning biosimilars, in 2020 Swiss pharmacists reported significantly more often a dispensing frequency of less than once a week compared to the German group (2020: ≥ 2 times a week: DE: 44.0% vs CH: 9.7%; ≤ 1 time a week: DE: 56.0% vs CH: 90.3%, *p* < 0.01; see Fig. 1, panel B). This difference disappeared in 2022, with more Swiss pharmacists dispensing biosimilars more than twice a week (2022: ≥ 2 times a week: DE: 38.7% vs CH: 23.8%; ≤ 1 time a week: DE: 61.3% vs CH: 76.2%, ns). In Switzerland between 2020 and 2022, there was a significant increase in the dispensing frequency of biosimilars more than twice a week (from 9.7% to 23.8%, *p* < 0.01).

### Knowledge of the definition of biosimilars

Knowledge of the term biosimilars did not differ between countries in 2020 and 2022, with 41.7% (DE, *n* = 35) and 50.0% (CH, *n* = 31) of correct answers in 2020, and 49.3% (DE, *n* = 37) and 41.6% (CH, *n* = 42) of correct answers in 2022 (ns). The most frequent error was that a biosimilar is a generic biological (2020: DE: 54.8% (*n* = 46) vs CH: 45.2% (*n* = 28), ns; 2022: DE: 45.3% (*n* = 34) vs CH: 58.4% (*n* = 59), ns). None of the participants had never heard of biosimilars, and none believed a biosimilar to be a counterfeit copy. There was no relationship between knowing the definition of biosimilars and the dispensing frequency of biologicals or biosimilars (data not shown).

### Attitudes towards biosimilars

**Fig. 2:**
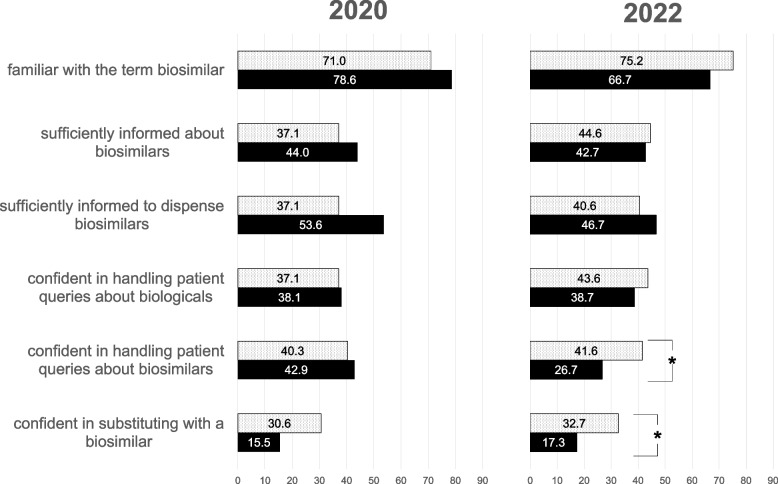
Agreement (in %) to 6 attitudes regarding biosimilars of Swiss (dotted bars) and German (solid bars) pharmacists regarding biosimilars in 2020 (left panel) and 2022 (right panel); statistical significance is marked with an asterisk

In 2020 and 2022, four out of six attitudes of German and Swiss participants remained unchanged that were: being familiar with the term biosimilar, feeling sufficiently informed about biosimilars and sufficiently informed to dispense biosimilars, and being confident in handling patient queries regarding a therapy with a biological (Fig. 2).

In 2022, the confidence in handling patient queries regarding a therapy with a biosimilar differed significantly between Swiss and German participants (2020: DE: 42.9% vs CH: 40.3%, ns; 2022: DE: 26.7% vs CH: 41.6%; *p* < 0.008) as well as the confidence in substituting with a biosimilar (Fig. 2) with Swiss participants indicating a higher confidence than German participants (2020: DE: 15.5% vs CH: 30.6%, ns; 2022: DE: 17.3% vs CH: 32.7%, *p* < 0.008).

### Knowledge on substitution rules and sources of information

In 2020 and 2022, at most one third of the participants in Germany and Switzerland were aware of the interdiction for a community pharmacist to substitute a biological with a biosimilar, which was the correct answer (2020: DE: 32.1% vs CH: 21.0%, ns; 2022: DE: 37.3% vs CH: 32.7%, ns). In 2020, significantly more Swiss pharmacists did not know that substitution was not allowed (2020: DE: 7.1% vs CH: 37.1%, *p* < 0.005), and significantly more German pharmacists thought it was allowed, but only for insulin products (2020: DE: 7.1% vs CH: 1.6%, *p* < 0.005). The difference disappeared in 2022 (2022: not allowed: DE: 16.0% vs CH: 24.8%, ns; only for insulin DE: 4.0% vs CH: 4.0%, ns).

A wide variety of information sources were named by the participants. The sources consulted once a week or more were primarily the Summary of Product Characteristics (SmPC)/Package leaflet (2020: DE: 40.5% vs CH: 35.5%, ns; 2022: DE: 40.0% vs CH: 35.6%, ns), followed by scientific publications (2020: DE: 26.2% vs CH: 21.0%, ns; 2022: DE: 24.0% vs CH: 13.9%, ns).

### Conditions for using biosimilars

**Fig. 3:**
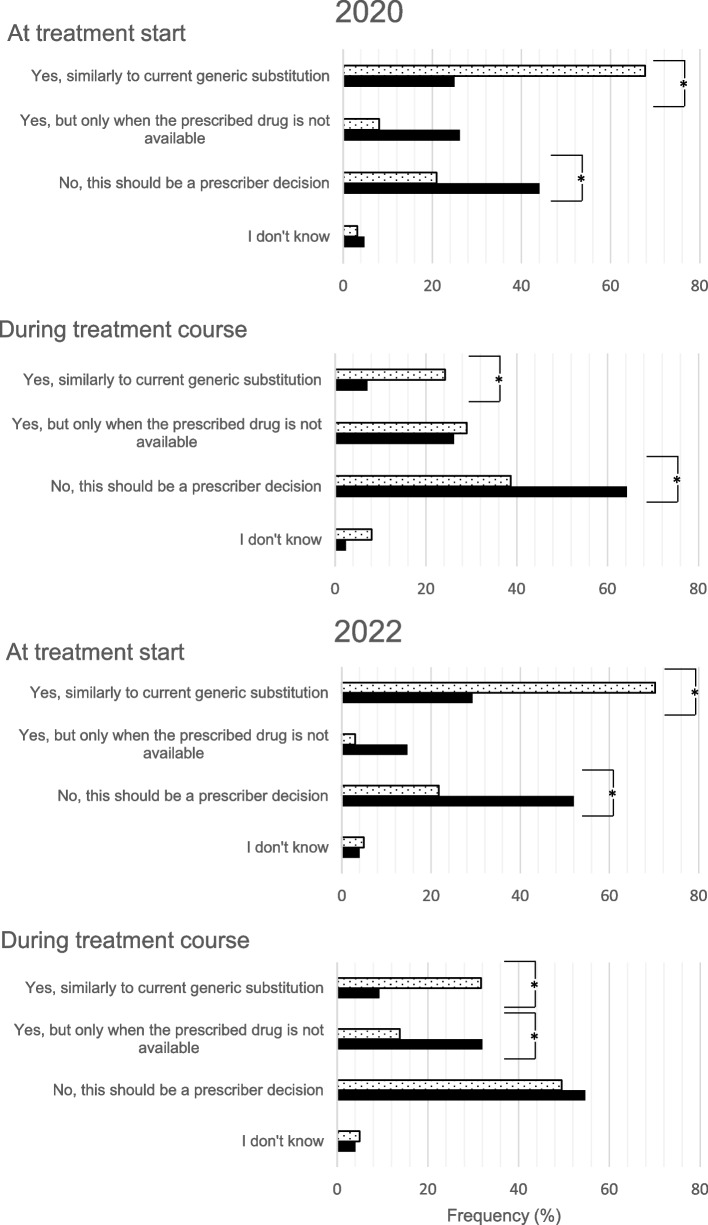
Comparison of answers on whether substitution should be permitted on treatment start or during treatment course by the Swiss (dotted bars) and German (solid bars) community pharmacists in 2020 (left panel) and 2022 (right panel); statistical significance is marked with an asterisk

In 2020 and 2022, German participants would predominantly refrain from substituting biologicals independently whether it is on treatment start (DE: 2020: 44.0% vs 2022: 52.0%, ns) or during treatment course (DE: 2020: 64.3% vs 2022: 54.7%, ns); for most of them, substitution should remain a prescribers’ decision (Fig. 3). Significantly more Swiss participants were of the opinion that substitution of a biological should be permitted for pharmacists at treatment start (2020: DE: 25.0% vs CH: 67.7%, *p* < 0.004; 2022: DE: 29.3% vs CH: 70.3%, *p* < 0.004; see Fig. 3). During treatment course, most of the Swiss participants stated that substitution should be a prescribers’ decision (Fig. 3). Substitution when the prescribed medicine is not available would be an option many German pharmacists would prefer (at treatment initiation: DE: 2020: 26.2% vs 2022: 14.7%, ns; during treatment course: DE: 2020: 26.2% vs 2022: 32.0%, ns; Fig. 3).

That biosimilars should never be used was rarely mentioned in 2020 and 2022 by German and Swiss participants but significantly more often by German participants (2020: DE: 6.0% vs CH: 1.6%, ns; 2022: DE: 10.7% vs CH: 0%, *p* < 0.004). The conditions for using biosimilars with the highest acceptance rates were the lowest price for Swiss pharmacists (2020: DE: 16.7% vs CH: 56.5%, *p* < 0.004; 2022: DE: 14.7% vs CH: 65.3%, *p* < 0.004) and when the originator medicine causes adverse drug reactions (ADR) for German pharmacists (2020: DE: 52.4% vs CH: 35.5%, ns; 2022: DE: 52.0% vs CH: 25.7%, *p* < 0.004).

### Training on biologicals

In 2020 and 2022, a comparable percentage of participants had obtained training on biologicals (2020: DE: 48.8% vs CH: 41.9%, ns; 2022: DE: 44.0% vs CH: 48.5%, ns) and was highly interested in additional training on this topic (2020: DE: 83.3% vs CH: 88.7%, ns; 2022: DE: 81.3% vs CH: 89.1%, ns). In both years and countries, there was no significant difference in giving the correct answer for the biosimilar definition between participants who had received any training on the topic of biologicals and participants without any training (2020: DE with training: 48.8% vs without training 32.5%, ns; CH with training: 42.3% vs without training 55.9%, ns; 2022: DE with training: 63.6% vs without training 42.1%, ns; CH with training: 42.9% vs without training 42.0%, ns).

In both years and independently of the country, knowing the substitution rules did not differ significantly between participants with and without training (2020: DE with training: 39.0% vs without training 25.0%, ns; CH with training: 23.1% vs without training 14.7%, ns; 2022: DE with training: 36.4% vs without training 39.5%, ns; CH with training: 44.9% vs without training 22.0%, ns).

### Influence of the COVID-19 pandemic

The opinion about the impact of the COVID-19 pandemic did not differ between German and Swiss participants. After two years of pandemic, the majority of the participating community pharmacists indicated a similar interest in biologicals and biosimilars (DE: 60.0% vs. CH: 55.5%; ns), similar working style with biologicals and biosimilars (DE: 56.0% vs CH: 65.4%; ns), and similar confidence in counselling on biologicals and biosimilars (DE: 61.3% vs. CH: 57.4%; ns). Approximately half of the participants were of the opinion that knowledge about biologicals and biosimilars remained unchanged before and after the pandemic (DE: 49.3% vs. CH: 53.5%; ns) as well as their feeling of readiness to assume more responsibility regarding biologicals and biosimilars in the future (DE: 45.3% vs CH: 52.5%; ns).

## Discussion

The results of this survey indicate a mixed picture of the knowledge and attitudes of German and Swiss pharmacists towards biologicals and biosimilars. Although almost three quarter of the participants responded that they werefamiliar with the term biosimilar, only half of them knew the correct definition of the term or were confident when it comes to counselling patients, and only one third of them knew the correct substitution rules in their own country. These results are in line with findings from other European countries such as France, where 77% of the pharmacists indicated “little knowledge” about biosimilars [25]. Even if knowledge does not predict behavior [33], and although we did not assess any correlates or determinants between knowledge and biosimilar substitution, it was unsurprising that only one third of the participants felt confident when substituting a biosimilar. The frequency of dispensing biosimilars significantly increased in Switzerland between 2020 and 2022 with more than twice as many participants who dispensed biologicals more than twice a week (from 9.7% to 23.8%). However, this number might not have greatly impacted the overall confidence of the Swiss pharmacists.

Overall, the similarities and differences in knowledge and perception between both countries remained unchanged before and after the COVID-19 pandemic, which leads us to the conclusion that in neither country the circumstances caused by the pandemic had a greater impact on changing pharmacists’ knowledge or attitudes toward biologicals and biosimilars than in the other. In both countries, the integration of new processes in the pharmacy due to the pandemic were challenging. As an example, the ordering, storage and distribution of COVID-19 vaccines were omnipresent. Other additional challenges such as production of disinfectant, distribution of FFP2 masks, managing drug shortages or lack of personnel intensified the crisis, and mental stress in general increased among pharmacists [34, 35]. Nevertheless, pharmacists indicated their general interest in biosimilars and their readiness to take more responsibilities in this field. Therefore, pharmacists seem to be receptive to an emerging unknown area. It is likely that pharmacists in both countries—although interested—had no capacities for additional training or self-study on topics such as biosimilars.

Another contributing factor to the persisting knowledge gaps might be the under representation of the topic biologicals/biosimilars in the curricula of pharmacy students. This hypothesis gets strengthened by findings in a French survey where nearly 8 out of 10 pharmacy residents stated they had “no knowledge” or “little knowledge” related to biosimilar medicines and felt less informed about biosimilar medicines compared to their older counterparts working at the hospital [25] . Therefore, integrating educational lessons or training on biologicals with emphasis on the regulatory and practical aspects in handling biosimilars in the curricula of pharmacy students might be one strategy to improve knowledge on biosimilars in future pharmacists.

For already practicing pharmacists, educational material in a time-saving format should be provided e.g., by health authorities (Swissmedic in Switzerland and BfArM in Germany). Both institutions already offer training courses on various topics. In addition, the pharmaceutical industry could provide written information on their products to the pharmacists since the SmPC where the information source used by 35–40% of the participants. The pharmaceutical industry remains a main information source and was mentioned by over 70% of the pharmacists in the French survey [25].

A sizable number of German and Swiss pharmacists were still of the opinion that substitution of biosimilars should only be a prescriber’s decision. While substitution in other areas is common practice in German and Swiss pharmacies, such as the generic substitution, deferring the substitution to someone else indicates a reluctance to take responsibility regarding biosimilars. In this sense, German and Swiss pharmacists seem to differ. The higher willingness of Swiss participants to substitute biosimilars in the same way as the generic substitution may be caused by a Swiss law that gives more freedom in substitution to Swiss pharmacists compared to German pharmacists [36]. In contrast to German pharmacists, Swiss pharmacists are not bound to contracts with health insurance companies for generic substitution. Notwithstanding, many pharmacists commented in our survey that a primary condition for biosimilar prescription and therefore for increasing dispense is the physicians' confidence and acceptance in biosimilars. Finally, controversial discussions about the permission to substitute or not, when they are placed in the public domain, may increase hesitancy and doubt of healthcare professionals [37].

In spite of all these hesitancies, about half of the participants maintained a positive attitude toward substituting a biological/biosimilar and a readiness to assume more responsibility regarding biologicals and biosimilars in the future. Thus, education and training represent the main solution to fill this gap, which was acknowledged by the participants with 85% of them wishing additional training on this topic.

For Switzerland, it is estimated that by the end of 2025, patent expirations will create a market for biosimilars that could compete with biologicals sales of CHF 500 million [38]. However, our research suggests that the topic of biosimilars was of less importance in German and Swiss pharmacies than would be necessary to fully exploit the financial advantages of biosimilars. To further improve the acceptance of biosimilars by patients and healthcare professionals, it is necessary to close the gaps in knowledge and understanding about biosimilars and their regulatory approval process. Two studies published in 2020 discussed possible approaches to address this. Concrete recommendations by European multi-stakeholders were for instance developing a clear and one-voice regulatory guidance about biosimilar interchangeability and switching across Europe, disseminate evidence from and experience with (multiple) switching, providing practical biosimilar product information and providing guidance about biosimilar use, and communicating the benefits provided by biosimilars and the introduction of market competition [24, 39]. Overall, next to price policies, awareness remains the main driver to increase uptake of biosimilars globally [40].

In addition, as is known from literature some practical barriers need to be overcome to increase biosimilars’ success [20]. One of the greatest practical barriers is potential patient concerns about their new medicine. Those concerns and the possible low expectations of the effect of the new medicine could lead to a nocebo effect [19, 20]. Furthermore, the switch in medication can lead to confusion and therefore unintended medication non-adherence [41].

In Germany since 2020, the automatic substitution of bioidenticals is mandatory due to established contracts with health insurance companies. A political decision is currently awaited to permit biosimilar substitution, which should come in force in August 2023. In view of such a change in pharmacy practice, training for pharmacists seems indispensable, especially because patients often react with negative attitudes when confronted with non-medical medication switches, that are defined as switches not motivated by a medical reason [41]. Thus, it is likely that the 85% survey participants who wished additional training on the topic biologicals/biosimilars were conscious about the imminent challenges in the healthcare systems in their countries.

In summary, the topic of biologicals/biosimilars should receive more attention. Our findings have implications on the future handling of biosimilars, their substitution in daily practice and the future training of pharmacists on this topic. As experts in this field, pharmacists should be able to competently address patients' concerns when initiating a therapy with a biological or switching a current biologic medicine to a biosimilar. Various barriers such as knowledge gaps especially in the substitution rules and an overall low confidence in handling were identified. Possible solutions were proposed based on those findings. Additional training is one of the most important and promising way to achieve an adequate level of expertise and confidence in pharmacists and is therefore required, among others.

This study had several strengths. First, the survey questions were identical in 2020 and 2022. Second, characteristics of all participants were similar in 2020 and 2022, allowing comparing the data. Third, even though newer biosimilars were approved between 2020 and 2022, and uptake was growing [42], there were no fundamental changes in substitution laws, nor breaking discoveries or news between 2020 and 2022 in the field of biologicals and biosimilar. The biosimilar landscapes in Germany and Switzerland were similar in 2020 and 2022 with most of the newly approved biosimilars being a further development of a biosimilar already on the market (e.g., adalimumab, bevacizumab, insulin aspart) [43]. Thus, the few influences on attitudes and knowledge that we have observed are likely due to fluctuation in the single respondents. Finally, 2023 is expected to be a turning point as new therapeutic areas will expand the use of biosimilars such as ophthalmology or gene therapy [44]. Thus, we claim that our results are robust and were not influenced by external factors.

Our study has some limitations. First, the survey was offered to Swiss pharmacists in German and English although four national languages exist. By doing so, Swiss pharmacists who speak neither German nor English were excluded in theory. However, we were able to recruit participants from all linguistic regions of the country. Even if non-German-speaking participants were a minority or underrepresented (38% in 2020 and 10% in 2022), this represents roughly the repartition of the languages over the Swiss territory with approximately 65% of German-speaking people in 2020. Thus, we claim that our results are generalizable to whole Switzerland. Second, the sample size was moderate, with 390 participants in 2020 and 374 participants in 2022. Nevertheless, we obtained full data sets from 50 to 69% of the participants, which demonstrates a large interest in the topic and is sufficient for statistical analyses [30, 31]. Third, we cannot exclude selection bias toward more interested, knowledgeable individuals who answered our surveys. Solutions to counteract and motivate unwilling individuals to participate have bias themselves such as unreliable answers with financial incentive. Thus, our results might overestimate the knowledge and interest of pharmacists in comparison to the overall Swiss and German pharmacist-population.

## Conclusion

Although the biosimilar market has grown continuously over the last years, there have been only limited changes in Swiss and German pharmacists' knowledge and attitudes. A lack of knowledge and confidence in dealing with biologicals is still present among the pharmacists, especially when it comes to substitution with biosimilars. It is likely that more responsibilities in this field are to be placed on pharmacists in the future, as we currently see in Germany and Norway with an ongoing discussion regarding permission for (automatic) substitution of biosimilars. Thus, issues such as hesitancy and misconception must be overcome. Therefore, actions must be taken such as promoting pharmacists’ confidence in dealing with biosimilars through individual training, among others.

### Supplementary Information

**Additional file 1.**

**Additional file 2.**

## Data Availability

The data generated during the study are not publicly available. Raw data are available from the corresponding author on reasonable request to ensure that they are only used for reasonable purposes. Raw data are stored in a secured digital environment.
