# Supplementary material for: Knowledge and attitudes of German and Swiss community pharmacists towards biologicals and biosimilars – a prospective survey before and after the COVID-19 pandemic
Source: BMC Health Serv Res. 2023 Dec 18;23:1432. doi: 10.1186/s12913-023-10475-x (PMC10726545; doi:10.1186/s12913-023-10475-x)
Supplement: Supplementary file 1 — Additional file 1. [file 12913_2023_10475_MOESM1_ESM.pdf]

Data Dictionary Codebook

12-09-2023 16:19

#	Variable / Field Name	Field Label<br>*Field Note*	Field Attributes (Field Type, Validation, Choices, Calculations, etc.)
**Instrument: Survey_Germany (survey_germany) Enabled as survey**			
1	[record_id]	Record ID	text
2	[participant_id]	Participant ID	text
3	[desc_einleitung]	Sehr geehrte Apothekerin, sehr geehrter ApothekerSehr geehrte Kollegin, sehr geehrter Kollege Als Apotheker*in in einer öffentlichen Apotheke sind Ihre Ansichten und Einstellungen zu Biologika und Biosimilars im Mittelpunkt unseres Interesses. Unsere kurze Umfrage (8 Minuten) untersucht die Meinungen, die Ansichten und den Informationsbedarf der Apotheker·innen zu Biologika und deren Substitution. Dies ist eine internationale Studie in Deutschland und der Schweiz. In Deutschland wird diese Untersuchung vom Geschäftsbereich Arzneimittel der ABDA - Bundesvereinigung Deutscher Apothekerverbände durchgeführt. Die von Ihnen bereitgestellten Informationen werden dazu beitragen, den Wissensstand über die Ansichten der Apotheker·innen zu Biologika in verschiedenen Ländern zu ermitteln. Ihre Teilnahme ist freiwillig. Ihre Antworten werden anonym erfasst und gemäss der nationalen Datenschutzbestimmung behandelt.	descriptive
4	[einverstaendnis]	Hiermit erkläre ich, dass ich die oben genannten Informationen gelesen und verstanden habe und freiwillig teilnehme. Ich gebe die Erlaubnis, dass meine Antworten erfasst und von den Forschenden in nationalen und internationalen Forschungsgruppen analysiert werden dürfen.	radio, Required<br>1 Ja<br>0 Nein
5	[desc_hi]<br>Show the field ONLY if:<br>[einverstaendnis] = '1'	Section Header:<br>Hintergrundinformationen	descriptive
6	[geschlecht]<br>Show the field ONLY if:<br>[einverstaendnis] = '1'	Was ist Ihr Geschlecht?	radio, Required<br>1 männlich<br>0 weiblich
7	[alter]<br>Show the field ONLY if:<br>[einverstaendnis] = '1'	Wie alt sind Sie?<br>*in Jahren*	text (integer, Min: 15, Max: 100), Required
8	[arbeitsjahre]<br>Show the field ONLY if:<br>[einverstaendnis] = '1'	Seit wie vielen Jahren sind Sie als Apotheker*in tätig?<br>*in Jahren*	text (integer, Min: 0, Max: 70), Required
9	[arbeitsbereich]<br>Show the field ONLY if:<br>[einverstaendnis] = '1'	In welchem Arbeitsbereich sind Sie zurzeit tätig? (Bei mehreren Stellen, wählen Sie bitte den Bereich mit dem höchsten Beschäftigungsgrad aus)	radio, Required<br>0 Offizin-Apotheke<br>1 Krankenhausapotheke<br>2 Regierung / Zulassungsbehörden<br>3 Universität<br>4 Pharmaindustrie oder Grosshandelsunternehmen<br>5 Ich bin nicht berufstätig<br>6 Anderer
10	[anderer_arbeitsbereich]<br>Show the field ONLY if:<br>[arbeitsbereich] = '6'	Anderer, welcher?	text, Required

11	[desc_abgabe]<br><br>Show the field ONLY if:<br>[arbeitsbereich] = '0' or [arbeitsbereich] = '1' or [arbeitsbereich] = '2' or [arbeitsbereich] = '4' or [arbeitsbereich] = '3' or [arbeitsbereich] = '5' or ([arbeitsbereich] = '6' and [anderer_arbeitsbereich] <> '')	Section Header:<br>Die nächsten 2 Fragen befassen sich mit der Abgabe von Biologika. In diesem Fragebogen bezieht sich der Begriff Biologika auf Produkte, die biotechnologisch in lebenden Systemen hergestellt werden. Biologika können biologische Originalpräparate oder Biosimilars sein.	descriptive
12	[abgabe_biol]<br><br>Show the field ONLY if:<br>[arbeitsbereich] = '0' or [arbeitsbereich] = '1' or [arbeitsbereich] = '2' or [arbeitsbereich] = '4' or [arbeitsbereich] = '3' or [arbeitsbereich] = '5' or ([arbeitsbereich] = '6' and [anderer_arbeitsbereich] <> '')	Wie oft geben Sie durchschnittlich Biologika ab?	radio, Required<br>0 Täglich oder mehrmals täglich<br>1 2 bis 6 Mal pro Woche<br>2 1 Mal pro Woche<br>3 Weniger als 1 Mal pro Woche<br>4 Nie<br><br>Question number: 1
13	[abgabe_sim]<br><br>Show the field ONLY if:<br>[arbeitsbereich] = '0' or [arbeitsbereich] = '1' or [arbeitsbereich] = '2' or [arbeitsbereich] = '4' or [arbeitsbereich] = '3' or [arbeitsbereich] = '5' or ([arbeitsbereich] = '6' and [anderer_arbeitsbereich] <> '')	Wie oft geben Sie durchschnittlich Biosimilars ab?	radio, Required<br>0 Täglich oder mehrmals täglich<br>1 2 bis 6 Mal pro Woche<br>2 1 Mal pro Woche<br>3 Weniger als 1 Mal pro Woche<br>4 Nie<br><br>Question number: 2
14	[desc_einstellung]<br><br>Show the field ONLY if:<br>[abgabe_sim] <> ''	Section Header:<br>Der nächste Teil des Fragebogens befasst sich mit Ihrer Einstellung gegenüber Biosimilars.	descriptive
15	[verstaendnis_sim]<br><br>Show the field ONLY if:<br>[abgabe_sim] <> ''	Welche Aussage beschreibt am besten, was Sie unter einem Biosimilar verstehen? Bitte wählen Sie nur eine Antwort aus.	radio, Required<br>0 Ein neues Biologikum<br>1 Ein generisches Biologikum<br>2 Eine gefälschte Kopie eines Biologikums<br>3 Eine ähnliche Kopie eines Biologikums<br>4 Ich habe von Biosimilars gehört, bin aber nicht sicher, was es ist<br>5 Ich habe noch nie von Biosimilars gehört<br><br>Question number: 3
16	[desc_matrix_einstellung]<br><br>Show the field ONLY if:<br>[abgabe_sim] <> ''	Wählen Sie bitte für jede der folgenden Aussagen diejenige Option, die Ihre Meinung am besten beschreibt.	descriptive<br>Question number: 4
17	[einstellung1]<br><br>Show the field ONLY if:<br>[abgabe_sim] <> ''	1) Ich bin vertraut mit dem Begriff Biosimilar	radio (Matrix), Required<br>0 Stimme voll zu<br>1 Stimme zu<br>2 Stimme weder zu noch nicht zu<br>3 Stimme nicht zu<br>4 Stimme überhaupt nicht zu
18	[einstellung2]<br><br>Show the field ONLY if:<br>[abgabe_sim] <> ''	2) Ich fühle mich genügend informiert über Biosimilars	radio (Matrix), Required<br>0 Stimme voll zu<br>1 Stimme zu<br>2 Stimme weder zu noch nicht zu<br>3 Stimme nicht zu<br>4 Stimme überhaupt nicht zu

19	[einstellung3]<br>Show the field ONLY if:<br>[abgabe_sim] <> ''	3) Ich fühle mich genügend informiert, um Patient*innen Biosimilars abzugeben	radio (Matrix), Required<br>0 Stimme voll zu<br>1 Stimme zu<br>2 Stimme weder zu noch nicht zu<br>3 Stimme nicht zu<br>4 Stimme überhaupt nicht zu
20	[einstellung4]<br>Show the field ONLY if:<br>[abgabe_sim] <> ''	4) Ich bin sicher im Umgang mit Fragen von Patient*innen bezüglich ihrer biologischen Therapie	radio (Matrix), Required<br>0 Stimme voll zu<br>1 Stimme zu<br>2 Stimme weder zu noch nicht zu<br>3 Stimme nicht zu<br>4 Stimme überhaupt nicht zu
21	[einstellung5]<br>Show the field ONLY if:<br>[abgabe_sim] <> ''	5) Ich fühle mich wohl, wenn ich Patient*innen den Nutzen und die Risiken von Biosimilars erkläre	radio (Matrix), Required<br>0 Stimme voll zu<br>1 Stimme zu<br>2 Stimme weder zu noch nicht zu<br>3 Stimme nicht zu<br>4 Stimme überhaupt nicht zu
22	[einstellung6]<br>Show the field ONLY if:<br>[abgabe_sim] <> ''	6) Ich fühle mich wohl bei der Substitution eines Biologikums durch ein Biosimilar, in einer Situation, in der Substitution in der Apotheke erlaubt ist	radio (Matrix), Required<br>0 Stimme voll zu<br>1 Stimme zu<br>2 Stimme weder zu noch nicht zu<br>3 Stimme nicht zu<br>4 Stimme überhaupt nicht zu
23	[einstellung7]<br>Show the field ONLY if:<br>[abgabe_sim] <> ''	7) Ich fühle mich wohl bei der Substitution eines Biologikums durch ein Bioidentical, in einer Situation, in der Substitution in der Apotheke erlaubt ist	radio (Matrix), Required<br>0 Stimme voll zu<br>1 Stimme zu<br>2 Stimme weder zu noch nicht zu<br>3 Stimme nicht zu<br>4 Stimme überhaupt nicht zu
24	[desc_austausch]<br>Show the field ONLY if:<br>[einstellung7] <> ''	Section Header:<br>Bei den nächsten Fragen geht es um die Substitution und die Austauschbarkeit von Biologika. Eine Substitution eines Medikamentes findet statt, wenn ein*e Apotheker*in ein Medikament durch ein anderes ersetzt, ohne den verschreibenden Arzt oder die verschreibende Ärztin zu informieren oder um Einverständnis zu bitten.	descriptive
25	[bewilligung]<br>Show the field ONLY if:<br>[einstellung7] <> ''	Ist die Substitution von Biologika nach Ihrem aktuellen Wissensstand in ihrem Land erlaubt?	radio, Required<br>1 Ja<br>2 Ja, aber nur bei Insulinprodukten<br>0 Nein<br>3 Weiss nicht<br>Question number: 5
26	[subst_bb]<br>Show the field ONLY if:<br>[einstellung7] <> ''	Sollte Ihrer Meinung nach die Substitution von Biologika durch Apotheker*innen bei Behandlungsbeginn erlaubt sein?	radio, Required<br>1 Ja, ähnlich wie bei der aktuellen Generikasubstitution<br>2 Ja, aber nur, wenn das verschriebene Medikament nicht verfügbar ist<br>0 Nein, das sollte die Entscheidung des*der Verordner*in sein<br>3 Weiss nicht<br>Question number: 6

27	[subst_bv]<br>Show the field ONLY if:<br>[einstellung7] <> ''	Sollte Ihrer Meinung nach die Substitution von Biologika durch Apotheker*innen während des Behandlungsverlaufes von ein*er Patient*in erlaubt sein?	radio, Required<br>1 Ja, ähnlich wie bei der aktuellen Generikasubstitution<br>2 Ja, aber nur, wenn das verschriebene Medikament nicht verfügbar ist<br>0 Nein, das sollte die Entscheidung des*der Verordner*in sein<br>3 Weiss nicht<br>Question number: 7
28	[desc_matrix_verwendung]<br>Show the field ONLY if:<br>[einstellung7] <> ''	Wann sollten Biosimilars Ihrer Meinung nach verwendet werden? Wählen Sie bitte für jedes der folgenden Szenarien diejenige Option, die Ihre Meinung am besten beschreibt.	descriptive<br>Question number: 8
29	[verwendung1]<br>Show the field ONLY if:<br>[einstellung7] <> ''	1) Sie sollten nie verwendet werden	radio (Matrix), Required<br>1 Stimme zu<br>0 Stimme nicht zu<br>2 Bin unsicher
30	[verwendung2]<br>Show the field ONLY if:<br>[einstellung7] <> ''	2) Wenn das Biosimilar den tiefsten Preis hat	radio (Matrix), Required<br>1 Stimme zu<br>0 Stimme nicht zu<br>2 Bin unsicher
31	[verwendung3]<br>Show the field ONLY if:<br>[einstellung7] <> ''	3) Wenn das Original unwirksam ist	radio (Matrix), Required<br>1 Stimme zu<br>0 Stimme nicht zu<br>2 Bin unsicher
32	[verwendung4]<br>Show the field ONLY if:<br>[einstellung7] <> ''	4) Wenn das Original unerwünschte Wirkungen verursacht	radio (Matrix), Required<br>1 Stimme zu<br>0 Stimme nicht zu<br>2 Bin unsicher
33	[desc_infoquellen]<br>Show the field ONLY if:<br>[verwendung4] <> ''	Section Header:<br>Im folgenden Teil dieses Fragebogens geht es um Ihre Informationsquellen betreffend Biologika.	descriptive
34	[schulung]<br>Show the field ONLY if:<br>[verwendung4] <> ''	Haben Sie während Ihrer Berufstätigkeit eine Schulung zum Thema der Biologika besucht (z.B. Fortbildungskurse, Vorlesungen, Diskussionen, Symposien, etc.)?	radio, Required<br>1 Ja<br>0 Nein<br>2 Weiss nicht<br>Question number: 9
35	[zus_schulungen]<br>Show the field ONLY if:<br>[verwendung4] <> ''	Wären Sie an zusätzlichen Schulungen zum Thema der Biologika interessiert?	radio, Required<br>1 Ja<br>0 Nein<br>2 Weiss nicht<br>Question number: 10
36	[desc_matrix_infoquellen]<br>Show the field ONLY if:<br>[verwendung4] <> ''	Wie oft konsultieren Sie durchschnittlich jede der folgenden Informationsquellen betreffend Biologika? Wählen Sie bitte für jede der folgenden Quellen diejenige Option aus, die Ihre Meinung am besten beschreibt.	descriptive<br>Question number: 11
37	[quelle1]<br>Show the field ONLY if:<br>[verwendung4] <> ''	1) Fachinformation/ Patienteninformation	radio (Matrix), Required<br>0 Täglich oder mehrmals täglich<br>1 2 bis 6 Mal pro Woche<br>2 1 Mal pro Woche<br>3 Weniger als 1 Mal pro Woche<br>4 Nie

38	[quelle2]<br><br>Show the field ONLY if:<br>[verwendung4] <> ''	2) Fachkolleg*innen im Gesundheitswesen	radio (Matrix), Required<br>0 Täglich oder mehrmals täglich<br>1 2 bis 6 Mal pro Woche<br>2 1 Mal pro Woche<br>3 Weniger als 1 Mal pro Woche<br>4 Nie
39	[quelle3]<br><br>Show the field ONLY if:<br>[verwendung4] <> ''	3) Professionelle nicht-wissenschaftliche Publikationen	radio (Matrix), Required<br>0 Täglich oder mehrmals täglich<br>1 2 bis 6 Mal pro Woche<br>2 1 Mal pro Woche<br>3 Weniger als 1 Mal pro Woche<br>4 Nie
40	[quelle4]<br><br>Show the field ONLY if:<br>[verwendung4] <> ''	4) Guidelines von Gesundheitsinstitutionen (z.B. von einem Spital)	radio (Matrix), Required<br>0 Täglich oder mehrmals täglich<br>1 2 bis 6 Mal pro Woche<br>2 1 Mal pro Woche<br>3 Weniger als 1 Mal pro Woche<br>4 Nie
41	[quelle5]<br><br>Show the field ONLY if:<br>[verwendung4] <> ''	5) Pharmaindustrie (Marketing- oder Lehrmaterial und Weiterbildungsevents)	radio (Matrix), Required<br>0 Täglich oder mehrmals täglich<br>1 2 bis 6 Mal pro Woche<br>2 1 Mal pro Woche<br>3 Weniger als 1 Mal pro Woche<br>4 Nie
42	[quelle6]<br><br>Show the field ONLY if:<br>[verwendung4] <> ''	6) Patientenorganisationen	radio (Matrix), Required<br>0 Täglich oder mehrmals täglich<br>1 2 bis 6 Mal pro Woche<br>2 1 Mal pro Woche<br>3 Weniger als 1 Mal pro Woche<br>4 Nie
43	[quelle7]<br><br>Show the field ONLY if:<br>[verwendung4] <> ''	7) Gesundheits- und Zulassungsbehörden (z.B. Lehrmaterial, Public Assessment Reports)	radio (Matrix), Required<br>0 Täglich oder mehrmals täglich<br>1 2 bis 6 Mal pro Woche<br>2 1 Mal pro Woche<br>3 Weniger als 1 Mal pro Woche<br>4 Nie
44	[quelle8]<br><br>Show the field ONLY if:<br>[verwendung4] <> ''	8) Wissenschaftliche Publikationen	radio (Matrix), Required<br>0 Täglich oder mehrmals täglich<br>1 2 bis 6 Mal pro Woche<br>2 1 Mal pro Woche<br>3 Weniger als 1 Mal pro Woche<br>4 Nie
45	[quelle9]<br><br>Show the field ONLY if:<br>[verwendung4] <> ''	9a) Länderspezifische elektronische Informationsquelle	radio (Matrix), Required<br>0 Täglich oder mehrmals täglich<br>1 2 bis 6 Mal pro Woche<br>2 1 Mal pro Woche<br>3 Weniger als 1 Mal pro Woche<br>4 Nie
46	[beispiel_quelle9]<br><br>Show the field ONLY if:<br>[quelle9] = '0' or [quelle9] = '1' or [quelle9] = '2' or [quelle9] = '3'	9b) Fügen Sie bitte eine relevante elektronische Informationsquelle für Ihr Land an.	text, Required

47	[pandemie]<br>Show the field ONLY if:<br>[beispiel_quelle9] <> '' or [quelle9] = '4'	Section Header:<br>Im vorletzten Teil dieses Fragebogens geht es um den Einfluss der Corona-Pandemie im Bereich Biologika. Die letzten 2 Jahre waren für alle Beteiligten im Gesundheitssystem mit grossen Herausforderungen verbunden. Bitte wählen Sie, welche der folgenden Aussagen für Sie zutreffen. Heute, im Vergleich zu vor der Pandemie...	descriptive<br>Question number: 12
48	[einfluss1]<br>Show the field ONLY if:<br>[beispiel_quelle9] <> '' or [quelle9] = '4'	... ist mein Interesse an Biologicals/Biosimilars gestiegen	radio (Matrix), Required<br>1 Stimme voll zu<br>2 Stimme zu<br>3 Weder noch<br>4 Stimme nicht zu<br>5 Stimme überhaupt nicht zu
49	[einfluss2]<br>Show the field ONLY if:<br>[beispiel_quelle9] <> '' or [quelle9] = '4'	... ist mein Wissen über Biologicals/Biosimilars gestiegen	radio (Matrix), Required<br>1 Stimme voll zu<br>2 Stimme zu<br>3 Weder noch<br>4 Stimme nicht zu<br>5 Stimme überhaupt nicht zu
50	[einfluss3]<br>Show the field ONLY if:<br>[beispiel_quelle9] <> '' or [quelle9] = '4'	... hat sich meine Arbeitsweise im Bereich Biologicals/Biosimilars verändert	radio (Matrix), Required<br>1 Stimme voll zu<br>2 Stimme zu<br>3 Weder noch<br>4 Stimme nicht zu<br>5 Stimme überhaupt nicht zu
51	[einfluss4]<br>Show the field ONLY if:<br>[beispiel_quelle9] <> '' or [quelle9] = '4'	... ist meine Sicherheit in der Beratung mit Biologicals/Biosimilars gestiegen	radio (Matrix), Required<br>1 Stimme voll zu<br>2 Stimme zu<br>3 Weder noch<br>4 Stimme nicht zu<br>5 Stimme überhaupt nicht zu
52	[einfluss5]<br>Show the field ONLY if:<br>[beispiel_quelle9] <> '' or [quelle9] = '4'	... fühle ich mich bereit, in Zukunft mehr Verantwortung im Bereich Biologicals/Biosimilars zu übernehmen	radio (Matrix), Required<br>1 Stimme voll zu<br>2 Stimme zu<br>3 Weder noch<br>4 Stimme nicht zu<br>5 Stimme überhaupt nicht zu
53	[austausch]<br>Show the field ONLY if:<br>[einfluss5] <> ''	Section Header:<br>Im abschliessenden Teil dieses Fragebogens soll es um den Austausch von Biosimilars ab August 2022 gehen: Spätestens bis zum August 2022 soll der Gemeinsame Bundesausschuss über die Möglichkeiten zum Austausch von ärztlich verordneten Biologika in Apotheken beschließen. Der derzeit diskutierte Entwurf sieht vor, dass die Apotheken dann zu einem Austausch bei Verordnung biotechnologisch hergestellter Arzneimittel verpflichtet sein werden. Bitte wählen Sie, welche der folgenden Aussagen für Sie zutreffen: Wenn Apotheken zukünftig einen Austausch von Biosimilars aufgrund von Rabattverträgen vornehmen sollen...	descriptive<br>Question number: 13
54	[austausch1]<br>Show the field ONLY if:<br>[einfluss5] <> ''	... begrüße ich das grundsätzlich.	radio (Matrix), Required<br>1 Stimme zu<br>2 Stimme eher zu<br>3 Stimme eher nicht zu<br>4 Stimme nicht zu<br>5 Weiß nicht

55	[austausch2]<br>Show the field ONLY if:<br>[einfluss5] <> ''	... finde ich positiv, dass Apotheken hier mehr Verantwortung übernehmen können.	radio (Matrix), Required<br>1 Stimme zu<br>2 Stimme eher zu

			3 Stimme eher nicht zu<br>4 Stimme nicht zu<br>5 Weiß nicht
56	[austausch3]<br>Show the field ONLY if:<br>[einfluss5] <> "	... erwarte ich Konflikte mit den verordnenden Ärzt*innen.	radio (Matrix), Required<br>1 Stimme zu<br>2 Stimme eher zu<br>3 Stimme eher nicht zu<br>4 Stimme nicht zu<br>5 Weiß nicht
57	[austausch4]<br>Show the field ONLY if:<br>[einfluss5] <> "	... erwarte ich Konflikte mit den Patient*innen.	radio (Matrix), Required<br>1 Stimme zu<br>2 Stimme eher zu<br>3 Stimme eher nicht zu<br>4 Stimme nicht zu<br>5 Weiß nicht
58	[austausch5]<br>Show the field ONLY if:<br>[einfluss5] <> "	... sehe ich ein Risiko für die Therapietreue (Adhärenz).	radio (Matrix), Required<br>1 Stimme zu<br>2 Stimme eher zu<br>3 Stimme eher nicht zu<br>4 Stimme nicht zu<br>5 Weiß nicht
59	[austausch6]<br>Show the field ONLY if:<br>[einfluss5] <> "	... halte ich es für erforderlich, dass der Arbeitsaufwand durch zusätzliche Beratungen, z. B. aufgrund anderer Darreichungsformen oder Hilfsmittel, gesondert honoriert wird.	radio (Matrix), Required<br>1 Stimme zu<br>2 Stimme eher zu<br>3 Stimme eher nicht zu<br>4 Stimme nicht zu<br>5 Weiß nicht
60	[austausch7]<br>Show the field ONLY if:<br>[einfluss5] <> "	... halte ich es für erforderlich, dass der Dokumentations- und Arbeitsaufwand für die Information des Arztes über das an den Patienten abgegebene Arzneimittel (ggf. inkl. der Chargennummer) gesondert honoriert wird.	radio (Matrix), Required<br>1 Stimme zu<br>2 Stimme eher zu<br>3 Stimme eher nicht zu<br>4 Stimme nicht zu<br>5 Weiß nicht
61	[kommentar]<br>Show the field ONLY if:<br>[austausch7] <> "	Section Header:<br>Möchten Sie gerne weitere Ansichten oder Erfahrungen zum Thema der Biologika und Biosimilars und deren Substitution mitteilen? Sie können ausserdem diese Umfrage im Allgemeinen kommentieren.	notes<br>Custom alignment: LH
62	[email_adresse]<br>Show the field ONLY if:<br>[austausch7] <> "	Wenn Sie Informationen zu den Resultaten dieser Studie erhalten möchten, dann geben Sie bitte ihre Email-Adresse an. Ihre Antworten können nicht mit dieser Email-Adresse in Verbindung gebracht werden.	text (email), Identifier
63	[desc_abschluss]<br>Show the field ONLY if:<br>[austausch7] <> "	Vielen Dank für Ihre Teilnahme, übermitteln Sie jetzt Ihre Antworten mit einem Klick auf Submit.	descriptive
64	[survey_germany_complete]	Section Header: *Form Status*<br>Complete?	dropdown<br>0 Incomplete<br>1 Unverified<br>2 Complete
