# Supplementary material for: Knowledge and attitudes of German and Swiss community pharmacists towards biologicals and biosimilars – a prospective survey before and after the COVID-19 pandemic
Source: BMC Health Serv Res. 2023 Dec 18;23:1432. doi: 10.1186/s12913-023-10475-x (PMC10726545; doi:10.1186/s12913-023-10475-x)
Supplement: Supplementary file 2 — Additional file 2. [file 12913_2023_10475_MOESM2_ESM.pdf]

# Data Dictionary Codebook

12-09-2023 16:20

| # | Variable / Field Name | Field Label<br>*Field Note* | Field Attributes (Field Type, Validation, Choices, Calculations, etc.) |
|---|---|---|---|
| **Instrument: Survey_Schweiz** (survey_schweiz) | **Enabled as survey** | | |
| 1 | [participant_id] | Participant ID | text |
| 2 | [participant_id_e84247] | Participant ID | text |
| 3 | [de_eng] | In welcher Sprache möchten Sie den Fragebogen ausfüllen?<br><br>--------------------------------------------------------------------------------<br><br>In which language do you want to fill in the questionnaire? | radio, Required<br>0 Deutsch<br>1 English |
| 4 | [desc_introduction]<br><br>Show the field ONLY if:<br>[de_eng] = '1' | Section Header:<br>Dear Pharmacist, dear colleagueAs pharmacist working in community pharmacy, your thoughts and attitudes towards biologic medicines are at the centre of our interest. Our short questionnaire (8 minutes) investigates the perceptions, attitudes and information needs of pharmacists towards biologic medicines and their substitution. This survey is open in Switzerland and Germany. In Switzerland this research is being led in by the Pharmaceutical Care Research Group (PCRG) of the University of Basel. The information provided will contribute to increased knowledge about pharmacists' perceptions on biologic medicines. Your participation is voluntary. Answers will be registered anonymously and handled in accordance with the Data Protection Act of Switzerland. | descriptive |
| 5 | [agreement]<br><br>Show the field ONLY if:<br>[de_eng] = '1' | I hereby declare to have read and understood the information provided above and accept free-willingly to participate. I allow my response to be recorded and analyzed by the researchers both nationally and in the international research group. | yesno, Required<br>1 Yes<br>0 No |
| 6 | [desc_bi]<br><br>Show the field ONLY if:<br>[agreement] = '1' | Section Header:<br>Background Information | descriptive |
| 7 | [language]<br><br>Show the field ONLY if:<br>[agreement] = '1' | In which part of Switzerland do you work? | radio, Required<br>0 German-speaking<br>1 French-speaking<br>2 Italian-speaking<br>3 Rhaeto-Romanic speaking<br>4 Germany |
| 8 | [gender]<br><br>Show the field ONLY if:<br>[agreement] = '1' | What is your gender? | radio, Required<br>1 male<br>0 female |
| 9 | [age]<br><br>Show the field ONLY if:<br>[agreement] = '1' | What is your age?<br>*in years* | text (integer, Min: 15, Max: 100), Required |
| 10 | [wy]<br><br>Show the field ONLY if:<br>[agreement] = '1' | For how many years have you been working as a pharmacist?<br>*in years* | text (integer, Min: 0, Max: 70), Required |
| 11 | [work_sect]<br><br>Show the field ONLY if:<br>[agreement] = '1' | In which working sector are you currently employed? (In case of multiple jobs, please indicate the sector with the highest degree of employment.) | radio, Required<br>0 Community pharmacy<br>1 Hospital pharmacy<br>2 Government / regulatory agencies<br>3 University<br>4 Pharmaceutical companies or wholesale company<br>5 I am not in working life<br>6 Other |

| | | | |
|---|---|---|---|
| 12 | [other_work_sect]<br><br>Show the field ONLY if:<br>[work_sect] = '6' | Please specify "Other". | text, Required |
| 13 | [work_pos]<br><br>Show the field ONLY if:<br>([work_sect] = '0' or [work_sect] = '1') AND [language] <> 4 | In which position in the pharmacy are you currently employed? | radio, Required<br>0 Pharmacist with a 3-4 year degree (BSc or equivalent)<br>1 Pharmacist with a 5 year degree (MSc or equivalent)<br>4 Pharmacist with a licence to practise<br>2 Pharmacy student<br>3 Other |
| 14 | [other_work_pos]<br><br>Show the field ONLY if:<br>[work_pos] = '3' | Please specify "Other". | text, Required |
| 15 | [desc_disp]<br><br>Show the field ONLY if:<br>([work_sect] = '2' or [work_sect] = '3' or [work_sect] = '4' or [work_sect] = '5' or ([work_sect] = '6' and [other_work_sect] <> '')) or ([work_pos] = '0' or [work_pos] = '1' or [work_pos] = '2' or ([work_pos] = '3' and [other_work_pos] <>'') or [work_pos] = '4') or ([language] = '4' AND ([work_sect] = '0' or [work_sect] ='1')) | Section Header:<br>The next two questions are about dispensing biologicals. In this questionnaire biologicals refer to products that are biotechnologically produced in living systems. Biologicals can be originator biologic products or biosimilars. | descriptive |
| 16 | [disp_biol]<br><br>Show the field ONLY if:<br>([work_sect] = '2' or [work_sect] = '3' or [work_sect] = '4' or [work_sect] = '5' or ([work_sect] = '6' and [other_work_sect] <> '')) or ([work_pos] = '0' or [work_pos] = '1' or [work_pos] = '2' or ([work_pos] = '3' and [other_work_pos] <>'') or [work_pos] = '4') or ([language] = '4' AND ([work_sect] = '0' or [work_sect] ='1')) | On average, how often do you dispense biologicals? | radio, Required<br>0 Every day or multiple times a day<br>1 2 to 6 times a week<br>2 Once a week<br>3 Less than once a week<br>4 Never<br><br>Question number: 1 |
| 17 | [disp_sim]<br><br>Show the field ONLY if:<br>([work_sect] = '2' or [work_sect] = '3' or [work_sect] = '4' or [work_sect] = '5' or ([work_sect] = '6' and [other_work_sect] <> '')) or ([work_pos] = '0' or [work_pos] = '1' or [work_pos] = '2' or ([work_pos] = '3' and [other_work_pos] <>'') or [work_pos] = '4') or ([language] = '4' AND ([work_sect] = '0' or [work_sect] ='1')) | On average, how often do you dispense biosimilars? | radio, Required<br>0 Every day or multiple times a day<br>1 2 to 6 times a week<br>2 Once a week<br>3 Less than once a week<br>4 Never<br><br>Question number: 2 |
| 18 | [desc_att]<br><br>Show the field ONLY if:<br>[disp_sim] <> '' | Section Header:<br>This next part is about your attitude towards biosimilars. | descriptive |
| 19 | [definition_sim]<br><br>Show the field ONLY if:<br>[disp_sim] <> '' | Which statement best describes what you understand a biosimilar to be? Please select one answer. | radio, Required<br>0 A new biological<br>1 A generic biological<br>2 A counterfeit copy of a biological<br>3 A similar copy of a biological<br>4 I have heard about biosimilars, but I am not sure what they are<br>5 I have never heard about biosimilars<br><br>Question number: 3 |

| | | | |
|---|---|---|---|
| 20 | [desc_matrix_att]<br><br>Show the field ONLY if:<br>[disp_sim] <> '' | For each of the following statement, please select one option which best describes your opinion. | descriptive<br>Question number: 4 |
| 21 | [attitude1]<br><br>Show the field ONLY if:<br>[disp_sim] <> '' | 1) I am familiar with the term biosimilar | radio (Matrix), Required<br>0 Strongly agree<br>1 Agree<br>2 Neither agree nor disagree<br>3 Disagree<br>4 Strongly disagree |
| 22 | [attitude2]<br><br>Show the field ONLY if:<br>[disp_sim] <> '' | 2) I feel sufficiently informed about biosimilars | radio (Matrix), Required<br>0 Strongly agree<br>1 Agree<br>2 Neither agree nor disagree<br>3 Disagree<br>4 Strongly disagree |
| 23 | [attitude3]<br><br>Show the field ONLY if:<br>[disp_sim] <> '' | 3) I feel sufficiently informed to dispense biosimilars to patients | radio (Matrix), Required<br>0 Strongly agree<br>1 Agree<br>2 Neither agree nor disagree<br>3 Disagree<br>4 Strongly disagree |
| 24 | [attitude4]<br><br>Show the field ONLY if:<br>[disp_sim] <> '' | 4) I am confident in handling patient queries regarding their biologic therapy | radio (Matrix), Required<br>0 Strongly agree<br>1 Agree<br>2 Neither agree nor disagree<br>3 Disagree<br>4 Strongly disagree |
| 25 | [attitude5]<br><br>Show the field ONLY if:<br>[disp_sim] <> '' | 5) I am comfortable explaining the benefit and risk of biosimilars to patients | radio (Matrix), Required<br>0 Strongly agree<br>1 Agree<br>2 Neither agree nor disagree<br>3 Disagree<br>4 Strongly disagree |
| 26 | [attitude6]<br><br>Show the field ONLY if:<br>[disp_sim] <> '' | 6) I am comfortable with substituting a biological medicine with a biosimilar, in a situation where substitution at the pharmacy is permitted | radio (Matrix), Required<br>0 Strongly agree<br>1 Agree<br>2 Neither agree nor disagree<br>3 Disagree<br>4 Strongly disagree |
| 27 | [attitude7]<br><br>Show the field ONLY if:<br>[disp_sim] <> '' AND [language] = '4' | 7) I am comfortable with substituting a biological medicine with a bioidentical, in a situation where substitution at the pharmacy is permitted | radio (Matrix), Required<br>0 Strongly agree<br>1 Agree<br>2 Neither agree nor disagree<br>3 Disagree<br>4 Strongly disagree |
| 28 | [desc_subst]<br><br>Show the field ONLY if:<br>[attitude6] <> '' | Section Header:<br>The next questions are about substitution and interchangeability of biologicals. Substitution of a medicine occurs when a pharmacist substitutes one drug for another without the prescribing physician's knowledge or consent. | descriptive |

| | | | |
|---|---|---|---|
| 29 | [permission]<br>Show the field ONLY if:<br>[attitude6] <> '' | To your current knowledge, is substitution of biologicals currently permitted in your country? | radio, Required<br>1 Yes<br>2 Yes, but only insulin products<br>0 No<br>3 Don't know<br>Question number: 5 |
| 30 | [subst_sot]<br>Show the field ONLY if:<br>[attitude6] <> '' | In your opinion, should substitution of a biological by a pharmacist be permitted on treatment initiation? | radio, Required<br>1 Yes, similarly to current generic substitution<br>2 Yes, but only when the prescribed drug is not available<br>0 No, this should be a prescriber decision<br>3 Don't know<br>Question number: 6 |
| 31 | [subst_dtc]<br>Show the field ONLY if:<br>[attitude6] <> '' | In your opinion, should substitution of a biological by a pharmacist be permitted during a patient's treatment course? | radio, Required<br>1 Yes, similarly to current generic substitution<br>2 Yes, but only when the prescribed drug is not available<br>0 No, this should be a prescriber decision<br>3 Don't know<br>Question number: 7 |
| 32 | [desc_matrix_use]<br>Show the field ONLY if:<br>[attitude6] <> '' | In your opinion, when should biosimilars be used? For each of the following scenarios, select the option that best describes your opinion. | descriptive<br>Question number: 8 |
| 33 | [use1]<br>Show the field ONLY if:<br>[attitude6] <> '' | 1) They should never be used | radio (Matrix), Required<br>1 Agree<br>0 Disagree<br>2 Uncertain |
| 34 | [use2]<br>Show the field ONLY if:<br>[attitude6] <> '' | 2) When the biosimilar has the lowest price | radio (Matrix), Required<br>1 Agree<br>0 Disagree<br>2 Uncertain |
| 35 | [use3]<br>Show the field ONLY if:<br>[attitude6] <> '' | 3) When the originator medicine is ineffective | radio (Matrix), Required<br>1 Agree<br>0 Disagree<br>2 Uncertain |
| 36 | [use4]<br>Show the field ONLY if:<br>[attitude6] <> '' | 4) When the originator medicine causes adverse reaction | radio (Matrix), Required<br>1 Agree<br>0 Disagree<br>2 Uncertain |
| 37 | [desc_info_sources]<br>Show the field ONLY if:<br>[use4] <> '' | Section Header:<br>The following part of the questionnaire is about your information sources on biologicals. | descriptive |
| 38 | [training]<br>Show the field ONLY if:<br>[use4] <> '' | In your working career, have you received any training on the topic of biologicals (e.g. continuous educational courses, lectures, discussions, symposiums etc.)? | radio, Required<br>1 Yes<br>0 No<br>2 Don't know<br>Question number: 9 |
| 39 | [add_training]<br>Show the field ONLY if:<br>[use4] <> '' | Would you be interested in receiving any additional training on the topic of biologicals? | radio, Required<br>1 Yes<br>0 No<br>2 Don't know<br>Question number: 10 |

| | | | |
|---|---|---|---|
| 40 | [desc_matrix_info]<br>Show the field ONLY if:<br>[use4] <> '' | On average, how often do you refer to each of the following information sources about biologicals? For each of the following, select the option that best describes your opinion. | descriptive<br>Question number: 11 |
| 41 | [source1]<br>Show the field ONLY if:<br>[use4] <> '' | 1) SmPC/package leaflet | radio (Matrix), Required<br>0 Every day or multiple times a day<br>1 2 to 6 times a week<br>2 Once a week<br>3 Less than once a week<br>4 Never |
| 42 | [source2]<br>Show the field ONLY if:<br>[use4] <> '' | 2) Fellow healthcare professionals | radio (Matrix), Required<br>0 Every day or multiple times a day<br>1 2 to 6 times a week<br>2 Once a week<br>3 Less than once a week<br>4 Never |
| 43 | [source3]<br>Show the field ONLY if:<br>[use4] <> '' | 3) Professional non-scientific publications | radio (Matrix), Required<br>0 Every day or multiple times a day<br>1 2 to 6 times a week<br>2 Once a week<br>3 Less than once a week<br>4 Never |
| 44 | [source4]<br>Show the field ONLY if:<br>[use4] <> '' | 4) Health institution guidelines (e.g. from a hospital) | radio (Matrix), Required<br>0 Every day or multiple times a day<br>1 2 to 6 times a week<br>2 Once a week<br>3 Less than once a week<br>4 Never |
| 45 | [source5]<br>Show the field ONLY if:<br>[use4] <> '' | 5) Pharmaceutical companies (marketing/ educational materials and educational events) | radio (Matrix), Required<br>0 Every day or multiple times a day<br>1 2 to 6 times a week<br>2 Once a week<br>3 Less than once a week<br>4 Never |
| 46 | [source6]<br>Show the field ONLY if:<br>[use4] <> '' | 6) Patient organisations | radio (Matrix), Required<br>0 Every day or multiple times a day<br>1 2 to 6 times a week<br>2 Once a week<br>3 Less than once a week<br>4 Never |
| 47 | [source7]<br>Show the field ONLY if:<br>[use4] <> '' | 7) Health authorities and regulatory agencies (e.g. educational materials, Public Assessment Reports etc.) | radio (Matrix), Required<br>0 Every day or multiple times a day<br>1 2 to 6 times a week<br>2 Once a week<br>3 Less than once a week<br>4 Never |
| 48 | [source8]<br>Show the field ONLY if:<br>[use4] <> '' | 8) Scientific publications | radio (Matrix), Required<br>0 Every day or multiple times a day<br>1 2 to 6 times a week<br>2 Once a week<br>3 Less than once a week<br>4 Never |

| | | | |
|---|---|---|---|
| 49 | [source9]<br>Show the field ONLY if:<br>[use4] <> '' | 9a) Country specific electronic information source | radio (Matrix), Required<br>0 Every day or multiple times a day<br>1 2 to 6 times a week<br>2 Once a week<br>3 Less than once a week<br>4 Never |
| 50 | [example_source9]<br>Show the field ONLY if:<br>[source9] = '0' or [source9] = '1' or [source9] = '2' or [source9] = '3' | 9b) Please add an electronic information source that is relevant for your country. | text, Required |
| 51 | [pandemic]<br>Show the field ONLY if:<br>[example_source9] <> '' or [source9] = '4' | Section Header:<br>This final part of the questionnaire is about the impact of the corona pandemic regarding biologicals. The last 2 years have been very challenging for all individuals involved in the healthcare system. For each of the following statements, please select the option that applies to you. Today, in comparison to before the pandemic... | descriptive<br>Question number: 12 |
| 52 | [impact1]<br>Show the field ONLY if:<br>[example_source9] <> '' or [source9] = '4' | ... my interest in biologicals/biosimilars has increased | radio (Matrix), Required<br>0 Strongly agree<br>1 Agree<br>2 Neither agree nor disagree<br>3 Disagree<br>4 Strongly disagree |
| 53 | [impact2]<br>Show the field ONLY if:<br>[example_source9] <> '' or [source9] = '4' | ... my knowledge about biologicals/biosimilars has increased | radio (Matrix), Required<br>0 Strongly agree<br>1 Agree<br>2 Neither agree nor disagree<br>3 Disagree<br>4 Strongly disagree |
| 54 | [impact3]<br>Show the field ONLY if:<br>[example_source9] <> '' or [source9] = '4' | ... my working style with biologicals/biosimilars has changed | radio (Matrix), Required<br>0 Strongly agree<br>1 Agree<br>2 Neither agree nor disagree<br>3 Disagree<br>4 Strongly disagree |
| 55 | [impact4]<br>Show the field ONLY if:<br>[example_source9] <> '' or [source9] = '4' | ... my confidence in counselling on biologicals/biosimilars has increased | radio (Matrix), Required<br>0 Strongly agree<br>1 Agree<br>2 Neither agree nor disagree<br>3 Disagree<br>4 Strongly disagree |
| 56 | [impact5]<br>Show the field ONLY if:<br>[example_source9] <> '' or [source9] = '4' | ... I feel ready to assume more responsibility regarding biologicals/biosimilars in the future | radio (Matrix), Required<br>0 Strongly agree<br>1 Agree<br>2 Neither agree nor disagree<br>3 Disagree<br>4 Strongly disagree |
| 57 | [comment_box]<br>Show the field ONLY if:<br>[impact5] <> '' | Section Header:<br>Would you like to convey further opinions and experiences about biologicals and biosimilars and the substitution of them? You can also comment this survey in general. | notes<br>Custom alignment: LH |
| 58 | [email_adress]<br>Show the field ONLY if:<br>[impact5] <> '' | If you want to be informed about the results of this study, please enter you email adress. The email address will not be associated with your answers. | text (email), Identifier |

| | | | |
|---|---|---|---|
| 59 | [desc_end]<br><br>Show the field ONLY if:<br>[impact5] <> '' | Thank you for your participation, please send us your responses by clicking the Submit button. | descriptive |
| 60 | [desc_einleitung]<br><br>Show the field ONLY if:<br>[de_eng] = '0' | Sehr geehrte Apothekerin, sehr geehrter ApothekerSehr geehrte Kollegin, sehr geehrter Kollege Als Apotheker·in in einer öffentlichen Apotheke sind Ihre Ansichten und Einstellungen zu Biologika und Biosimilars im Mittelpunkt unseres Interesses. Unsere kurze Umfrage (8 Minuten) untersucht die Meinungen, die Ansichten und den Informationsbedarf der Apotheker·innen zu Biologika und deren Substitution. Diese Umfrage ist offen in der Schweiz und Deutschland. In der Schweiz wird diese Untersuchung von der Pharmaceutical Care Research Group (PCRG) der Universität Basel durchgeführt. Die von Ihnen bereitgestellten Informationen werden dazu beitragen, den Wissensstand über die Ansichten der Apotheker·innen zu Biologika zu ermitteln. Ihre Teilnahme ist freiwillig. Ihre Antworten werden anonym erfasst und gemäss der nationalen Datenschutzbestimmung behandelt. | descriptive |
| 61 | [einverstaendnis]<br><br>Show the field ONLY if:<br>[de_eng] = '0' | Hiermit erkläre ich, dass ich die oben genannten Informationen gelesen und verstanden habe und freiwillig teilnehme. Ich gebe die Erlaubnis, dass meine Antworten erfasst und von den Forschenden in nationalen und internationalen Forschungsgruppen analysiert werden dürfen. | radio, Required<br>1 Ja<br>0 Nein |
| 62 | [desc_hi]<br><br>Show the field ONLY if:<br>[einverstaendnis] = '1' | Section Header:<br>Hintergrundinformationen | descriptive |
| 63 | [sprachregion]<br><br>Show the field ONLY if:<br>[einverstaendnis] = '1' | In welcher Sprachregion arbeiten Sie? | radio, Required<br>0 Deutschschweiz<br>1 Französische Schweiz<br>2 Italienische Schweiz<br>3 Rätoromanische Schweiz<br>4 Deutschland |
| 64 | [geschlecht]<br><br>Show the field ONLY if:<br>[einverstaendnis] = '1' | Was ist Ihr Geschlecht? | radio, Required<br>1 männlich<br>0 weiblich |
| 65 | [alter]<br><br>Show the field ONLY if:<br>[einverstaendnis] = '1' | Wie alt sind Sie?<br>*in Jahren* | text (integer, Min: 15, Max: 100), Required |
| 66 | [arbeitsjahre]<br><br>Show the field ONLY if:<br>[einverstaendnis] = '1' | Seit wie vielen Jahren sind Sie als Apotheker·in tätig?<br>*in Jahren* | text (integer, Min: 0, Max: 70), Required |
| 67 | [arbeitsbereich]<br><br>Show the field ONLY if:<br>[einverstaendnis] = '1' | In welchem Arbeitsbereich sind Sie zurzeit tätig? (Bei mehreren Stellen, wählen Sie bitte den Bereich mit dem höchsten Beschäftigungsgrad aus) | radio, Required<br>0 Offizin-Apotheke<br>1 Spitalpharmazie / Krankenhausapotheke<br>2 Regierung / Zulassungsbehörden<br>3 Universität<br>4 Pharmaindustrie oder Grosshandelsunternehmen<br>5 Ich bin nicht berufstätig<br>6 Anderer |
| 68 | [anderer_arbeitsbereich]<br><br>Show the field ONLY if:<br>[arbeitsbereich] = '6' | Anderer, welcher? | text, Required |

| | | | |
|---|---|---|---|
| 69 | [anstellung]<br><br>Show the field ONLY if:<br>([arbeitsbereich] = '0' or [arbeitsbereich] = '1') AND [sprachregion] <> 4 | In welcher Stelle in der Apotheke sind Sie zurzeit angestellt? | radio, Required<br>0 Pharmazeut·in mit einem BSc oder gleichwertigen Abschluss<br>1 Pharmazeut·in mit einem MSc oder gleichwertigen Abschluss<br>4 Apotheker·in mit einer Berufsausübungsbewilligung (BAB)<br>2 Pharmaziestudent·in<br>3 Andere |
| 70 | [andere_position]<br><br>Show the field ONLY if:<br>[anstellung] = '3' | Andere, welche? | text, Required |
| 71 | [desc_abgabe]<br><br>Show the field ONLY if:<br>([arbeitsbereich] = '2' or [arbeitsbereich] = '3' or [arbeitsbereich] = '4' or [arbeitsbereich] = '5' or ([arbeitsbereich] = '6' and [anderer_arbeitsbereich] <>'')) or ([anstellung] = '0' or [anstellung] = '1' or [anstellung] = '2' or ([anstellung] = '3' and [andere_position] <>'') or [anstellung] = '4') or ([sprachregion] = '4' AND ([arbeitsbereich] = '0' or [arbeitsbereich] = '1')) | Section Header:<br>Die nächsten 2 Fragen befassen sich mit der Abgabe von Biologika. In diesem Fragebogen bezieht sich der Begriff Biologika auf Produkte, die biotechnologisch in lebenden Systemen hergestellt werden. Biologika können biologische Originalpräparate oder Biosimilars sein. | descriptive |
| 72 | [abgabe_biol]<br><br>Show the field ONLY if:<br>([arbeitsbereich] = '2' or [arbeitsbereich] = '3' or [arbeitsbereich] = '4' or [arbeitsbereich] = '5' or ([arbeitsbereich] = '6' and [anderer_arbeitsbereich] <>'')) or ([anstellung] = '0' or [anstellung] = '1' or [anstellung] = '2' or ([anstellung] = '3' and [andere_position] <>'') or [anstellung] = '4') or ([sprachregion] = '4' AND ([arbeitsbereich] = '0' or [arbeitsbereich] = '1')) | Wie oft geben Sie durchschnittlich Biologika ab? | radio, Required<br>0 Täglich oder mehrmals täglich<br>1 2 bis 6 Mal pro Woche<br>2 1 Mal pro Woche<br>3 Weniger als 1 Mal pro Woche<br>4 Nie<br><br>Question number: 1 |
| 73 | [abgabe_sim]<br><br>Show the field ONLY if:<br>([arbeitsbereich] = '2' or [arbeitsbereich] = '3' or [arbeitsbereich] = '4' or [arbeitsbereich] = '5' or ([arbeitsbereich] = '6' and [anderer_arbeitsbereich] <>'')) or ([anstellung] = '0' or [anstellung] = '1' or [anstellung] = '2' or ([anstellung] = '3' and [andere_position] <>'') or [anstellung] = '4') or ([sprachregion] = '4' AND ([arbeitsbereich] = '0' or [arbeitsbereich] = '1')) | Wie oft geben Sie durchschnittlich Biosimilars ab? | radio, Required<br>0 Täglich oder mehrmals täglich<br>1 2 bis 6 Mal pro Woche<br>2 1 Mal pro Woche<br>3 Weniger als 1 Mal pro Woche<br>4 Nie<br><br>Question number: 2 |
| 74 | [desc_einstellung]<br><br>Show the field ONLY if:<br>[abgabe_sim] <> '' | Section Header:<br>Der nächste Teil des Fragebogens befasst sich mit Ihrer Einstellung gegenüber Biosimilars. | descriptive |
| 75 | [verstaendnis_sim]<br><br>Show the field ONLY if:<br>[abgabe_sim] <> '' | Welche Aussage beschreibt am besten, was Sie unter einem Biosimilar verstehen? Bitte wählen Sie nur eine Antwort aus. | radio, Required<br>0 Ein neues Biologikum<br>1 Ein generisches Biologikum<br>2 Eine gefälschte Kopie eines Biologikums<br>3 Eine ähnliche Kopie eines Biologikums<br>4 Ich habe von Biosimilars gehört, bin aber nicht sicher, was es ist<br>5 Ich habe noch nie von Biosimilars gehört<br><br>Question number: 3 |

| | | | |
|---|---|---|---|
| 76 | [desc_matrix_einstellung]<br>Show the field ONLY if:<br>[abgabe_sim] <> '' | Wählen Sie bitte für jede der folgenden Aussagen diejenige Option, die Ihre Meinung am besten beschreibt. | descriptive<br>Question number: 4 |
| 77 | [einstellung1]<br>Show the field ONLY if:<br>[abgabe_sim] <> '' | 1) Ich bin vertraut mit dem Begriff Biosimilar | radio (Matrix), Required<br>0 Stimme voll zu<br>1 Stimme zu<br>2 Stimme weder zu noch nicht zu<br>3 Stimme nicht zu<br>4 Stimme überhaupt nicht zu |
| 78 | [einstellung2]<br>Show the field ONLY if:<br>[abgabe_sim] <> '' | 2) Ich fühle mich genügend informiert über Biosimilars | radio (Matrix), Required<br>0 Stimme voll zu<br>1 Stimme zu<br>2 Stimme weder zu noch nicht zu<br>3 Stimme nicht zu<br>4 Stimme überhaupt nicht zu |
| 79 | [einstellung3]<br>Show the field ONLY if:<br>[abgabe_sim] <> '' | 3) Ich fühle mich genügend informiert, um Patient·innen Biosimilars abzugeben | radio (Matrix), Required<br>0 Stimme voll zu<br>1 Stimme zu<br>2 Stimme weder zu noch nicht zu<br>3 Stimme nicht zu<br>4 Stimme überhaupt nicht zu |
| 80 | [einstellung4]<br>Show the field ONLY if:<br>[abgabe_sim] <> '' | 4) Ich bin sicher im Umgang mit Fragen von Patient·innen bezüglich ihrer biologischen Therapie | radio (Matrix), Required<br>0 Stimme voll zu<br>1 Stimme zu<br>2 Stimme weder zu noch nicht zu<br>3 Stimme nicht zu<br>4 Stimme überhaupt nicht zu |
| 81 | [einstellung5]<br>Show the field ONLY if:<br>[abgabe_sim] <> '' | 5) Ich fühle mich wohl, wenn ich Patient·innen den Nutzen und die Risiken von Biosimilars erkläre | radio (Matrix), Required<br>0 Stimme voll zu<br>1 Stimme zu<br>2 Stimme weder zu noch nicht zu<br>3 Stimme nicht zu<br>4 Stimme überhaupt nicht zu |
| 82 | [einstellung6]<br>Show the field ONLY if:<br>[abgabe_sim] <> '' | 6) Ich fühle mich wohl bei der Substitution eines Biologikums durch ein Biosimilar, in einer Situation, in der Substitution in der Apotheke erlaubt ist | radio (Matrix), Required<br>0 Stimme voll zu<br>1 Stimme zu<br>2 Stimme weder zu noch nicht zu<br>3 Stimme nicht zu<br>4 Stimme überhaupt nicht zu |
| 83 | [einstellung7]<br>Show the field ONLY if:<br>[abgabe_sim] <> '' and [sprachregion] = '4' | 7) Ich fühle mich wohl bei der Substitution eines Biologikums durch ein Bioidentical, in einer Situation, in der Substitution in der Apotheke erlaubt ist | radio (Matrix), Required<br>0 Stimme voll zu<br>1 Stimme zu<br>2 Stimme weder zu noch nicht zu<br>3 Stimme nicht zu<br>4 Stimme überhaupt nicht zu |
| 84 | [desc_austausch]<br>Show the field ONLY if:<br>[einstellung6] <> '' | Section Header:<br>Bei den nächsten Fragen geht es um die Substitution und die Austauschbarkeit von Biologika. Eine Substitution eines Medikamentes findet statt, wenn ein·e Apotheker·in ein Medikament durch ein anderes ersetzt, ohne den verschreibenden Arzt oder die verschreibende Ärztin zu informieren oder um Einverständnis zu bitten. | descriptive |

| | | | |
|---|---|---|---|
| 85 | [bewilligung]<br>Show the field ONLY if:<br>[einstellung6] <> '' | Ist die Substitution von Biologika nach Ihrem aktuellen Wissensstand in ihrem Land erlaubt? | radio, Required<br>1 Ja<br>2 Ja, aber nur bei Insulinprodukten<br>0 Nein<br>3 Weiss nicht<br>Question number: 5 |
| 86 | [subst_bb]<br>Show the field ONLY if:<br>[einstellung6] <> '' | Sollte Ihrer Meinung nach die Substitution von Biologika durch Apotheker·innen bei Behandlungsbeginn erlaubt sein? | radio, Required<br>1 Ja, ähnlich wie bei der aktuellen Generikasubstitution<br>2 Ja, aber nur, wenn das verschriebene Medikament nicht verfügbar ist<br>0 Nein, das sollte die Entscheidung des·der Verordner·in sein<br>3 Weiss nicht<br>Question number: 6 |
| 87 | [subst_bv]<br>Show the field ONLY if:<br>[einstellung6] <> '' | Sollte Ihrer Meinung nach die Substitution von Biologika durch Apotheker·innen während des Behandlungsverlaufes von ein·er Patient·in erlaubt sein? | radio, Required<br>1 Ja, ähnlich wie bei der aktuellen Generikasubstitution<br>2 Ja, aber nur, wenn das verschriebene Medikament nicht verfügbar ist<br>0 Nein, das sollte die Entscheidung des·der Verordner·in sein<br>3 Weiss nicht<br>Question number: 7 |
| 88 | [desc_matrix_verwendung]<br>Show the field ONLY if:<br>[einstellung6] <> '' | Wann sollten Biosimilars Ihrer Meinung nach verwendet werden? Wählen Sie bitte für jedes der folgenden Szenarien diejenige Option, die Ihre Meinung am besten beschreibt. | descriptive<br>Question number: 8 |
| 89 | [verwendung1]<br>Show the field ONLY if:<br>[einstellung6] <> '' | 1) Sie sollten nie verwendet werden | radio (Matrix), Required<br>1 Stimme zu<br>0 Stimme nicht zu<br>2 Bin unsicher |
| 90 | [verwendung2]<br>Show the field ONLY if:<br>[einstellung6] <> '' | 2) Wenn das Biosimilar den tiefsten Preis hat | radio (Matrix), Required<br>1 Stimme zu<br>0 Stimme nicht zu<br>2 Bin unsicher |
| 91 | [verwendung3]<br>Show the field ONLY if:<br>[einstellung6] <> '' | 3) Wenn das Original unwirksam ist | radio (Matrix), Required<br>1 Stimme zu<br>0 Stimme nicht zu<br>2 Bin unsicher |
| 92 | [verwendung4]<br>Show the field ONLY if:<br>[einstellung6] <> '' | 4) Wenn das Original unerwünschte Wirkungen verursacht | radio (Matrix), Required<br>1 Stimme zu<br>0 Stimme nicht zu<br>2 Bin unsicher |
| 93 | [desc_infoquellen]<br>Show the field ONLY if:<br>[verwendung4] <> '' | Section Header:<br>Im folgenden Teil dieses Fragebogens geht es um Ihre Informationsquellen betreffend Biologika. | descriptive |
| 94 | [schulung]<br>Show the field ONLY if:<br>[verwendung4] <> '' | Haben Sie während Ihrer Berufstätigkeit eine Schulung zum Thema der Biologika besucht (z.B. Fortbildungskurse, Vorlesungen, Diskussionen, Symposien, etc.)? | radio, Required<br>1 Ja<br>0 Nein<br>2 Weiss nicht<br>Question number: 9 |

| | | | |
|---|---|---|---|
| 95 | [zus_schulungen]<br>Show the field ONLY if:<br>[verwendung4] <> '' | Wären Sie an zusätzlichen Schulungen zum Thema der Biologika interessiert? | radio, Required<br>1 Ja<br>0 Nein<br>2 Weiss nicht<br>Question number: 10 |
| 96 | [desc_matrix_infoquellen]<br>Show the field ONLY if:<br>[verwendung4] <> '' | Wie oft konsultieren Sie durchschnittlich jede der folgenden Informationsquellen betreffend Biologika? Wählen Sie bitte für jede der folgenden Quellen diejenige Option aus, die Ihre Meinung am besten beschreibt. | descriptive<br>Question number: 11 |
| 97 | [quelle1]<br>Show the field ONLY if:<br>[verwendung4] <> '' | 1) Fachinformation/ Patienteninformation | radio (Matrix), Required<br>0 Täglich oder mehrmals täglich<br>1 2 bis 6 Mal pro Woche<br>2 1 Mal pro Woche<br>3 Weniger als 1 Mal pro Woche<br>4 Nie |
| 98 | [quelle2]<br>Show the field ONLY if:<br>[verwendung4] <> '' | 2) Fachkolleg·innen im Gesundheitswesen | radio (Matrix), Required<br>0 Täglich oder mehrmals täglich<br>1 2 bis 6 Mal pro Woche<br>2 1 Mal pro Woche<br>3 Weniger als 1 Mal pro Woche<br>4 Nie |
| 99 | [quelle3]<br>Show the field ONLY if:<br>[verwendung4] <> '' | 3) Professionelle nicht-wissenschaftliche Publikationen | radio (Matrix), Required<br>0 Täglich oder mehrmals täglich<br>1 2 bis 6 Mal pro Woche<br>2 1 Mal pro Woche<br>3 Weniger als 1 Mal pro Woche<br>4 Nie |
| 100 | [quelle4]<br>Show the field ONLY if:<br>[verwendung4] <> '' | 4) Guidelines von Gesundheitsinstitutionen (z.B. von einem Spital) | radio (Matrix), Required<br>0 Täglich oder mehrmals täglich<br>1 2 bis 6 Mal pro Woche<br>2 1 Mal pro Woche<br>3 Weniger als 1 Mal pro Woche<br>4 Nie |
| 101 | [quelle5]<br>Show the field ONLY if:<br>[verwendung4] <> '' | 5) Pharmaindustrie (Marketing- oder Lehrmaterial und Weiterbildungsevents) | radio (Matrix), Required<br>0 Täglich oder mehrmals täglich<br>1 2 bis 6 Mal pro Woche<br>2 1 Mal pro Woche<br>3 Weniger als 1 Mal pro Woche<br>4 Nie |
| 102 | [quelle6]<br>Show the field ONLY if:<br>[verwendung4] <> '' | 6) Patientenorganisationen | radio (Matrix), Required<br>0 Täglich oder mehrmals täglich<br>1 2 bis 6 Mal pro Woche<br>2 1 Mal pro Woche<br>3 Weniger als 1 Mal pro Woche<br>4 Nie |
| 103 | [quelle7]<br>Show the field ONLY if:<br>[verwendung4] <> '' | 7) Gesundheits- und Zulassungsbehörden (z.B. Lehrmaterial, Public Assessment Reports) | radio (Matrix), Required<br>0 Täglich oder mehrmals täglich<br>1 2 bis 6 Mal pro Woche<br>2 1 Mal pro Woche<br>3 Weniger als 1 Mal pro Woche<br>4 Nie |

| | | | |
|---|---|---|---|
| 104 | [quelle8]<br>Show the field ONLY if:<br>[verwendung4] <> '' | 8) Wissenschaftliche Publikationen | radio (Matrix), Required<br>0 Täglich oder mehrmals täglich<br>1 2 bis 6 Mal pro Woche<br>2 1 Mal pro Woche<br>3 Weniger als 1 Mal pro Woche<br>4 Nie |
| 105 | [quelle9]<br>Show the field ONLY if:<br>[verwendung4] <> '' | 9a) Länderspezifische elektronische Informationsquelle | radio (Matrix), Required<br>0 Täglich oder mehrmals täglich<br>1 2 bis 6 Mal pro Woche<br>2 1 Mal pro Woche<br>3 Weniger als 1 Mal pro Woche<br>4 Nie |
| 106 | [beispiel_quelle9]<br>Show the field ONLY if:<br>[quelle9] = '0' or [quelle9] = '1' or [quelle9] = '2' or [quelle9] = '3' | 9b) Fügen Sie bitte eine relevante elektronische Informationsquelle für Ihr Land an. | text, Required |
| 107 | [pandemie]<br>Show the field ONLY if:<br>[beispiel_quelle9] <> '' or [quelle9] = '4' | Section Header:<br>Im abschliessenden Teil dieses Fragebogens geht es um den Einfluss der Corona-Pandemie im Bereich Biologika.Die letzten 2 Jahre waren für alle Beteiligten im Gesundheitssystem mit grossen Herausforderungen verbunden. Bitte wählen Sie, welche der folgenden Aussagen für Sie zutreffen.Heute, im Vergleich zu vor der Pandemie... | descriptive<br>Question number: 12 |
| 108 | [einfluss1]<br>Show the field ONLY if:<br>[beispiel_quelle9] <> '' or [quelle9] = '4' | ... ist mein Interesse an Biologicals/Biosimilars gestiegen | radio (Matrix), Required<br>1 Stimme voll zu<br>2 Stimme zu<br>3 Weder noch<br>4 Stimme nicht zu<br>5 Stimme überhaupt nicht zu |
| 109 | [einfluss2]<br>Show the field ONLY if:<br>[beispiel_quelle9] <> '' or [quelle9] = '4' | ... ist mein Wissen über Biologicals/Biosimilars gestiegen | radio (Matrix), Required<br>1 Stimme voll zu<br>2 Stimme zu<br>3 Weder noch<br>4 Stimme nicht zu<br>5 Stimme überhaupt nicht zu |
| 110 | [einfluss3]<br>Show the field ONLY if:<br>[beispiel_quelle9] <> '' or [quelle9] = '4' | ... hat sich meine Arbeitsweise im Bereich Biologicals/Biosimilars verändert | radio (Matrix), Required<br>1 Stimme voll zu<br>2 Stimme zu<br>3 Weder noch<br>4 Stimme nicht zu<br>5 Stimme überhaupt nicht zu |
| 111 | [einfluss4]<br>Show the field ONLY if:<br>[beispiel_quelle9] <> '' or [quelle9] = '4' | ... ist meine Sicherheit in der Beratung mit Biologicals/Biosimilars gestiegen | radio (Matrix), Required<br>1 Stimme voll zu<br>2 Stimme zu<br>3 Weder noch<br>4 Stimme nicht zu<br>5 Stimme überhaupt nicht zu |
| 112 | [einfluss5]<br>Show the field ONLY if:<br>[beispiel_quelle9] <> '' or [quelle9] = '4' | ... fühle ich mich bereit, in Zukunft mehr Verantwortung im Bereich Biologicals/Biosimilars zu übernehmen | radio (Matrix), Required<br>1 Stimme voll zu<br>2 Stimme zu<br>3 Weder noch<br>4 Stimme nicht zu<br>5 Stimme überhaupt nicht zu |

| | | | |
|---|---|---|---|
| 113 | [kommentar]<br>Show the field ONLY if:<br>[einfluss5] <> '' | Section Header:<br>Möchten Sie gerne weitere Ansichten oder Erfahrungen zum Thema der Biologika und Biosimilars und deren Substitution mitteilen? Sie können ausserdem diese Umfrage im Allgemeinen kommentieren. | notes<br>Custom alignment: LH |
| 114 | [email_adresse]<br>Show the field ONLY if:<br>[einfluss5] <> '' | Wenn Sie Informationen zu den Resultaten dieser Studie erhalten möchten, dann geben Sie bitte ihre Email-Adresse an. Ihre Antworten können nicht mit dieser Email-Adresse in Verbindung gebracht werden. | text (email), Identifier |
| 115 | [desc_abschluss]<br>Show the field ONLY if:<br>[einfluss5] <> '' | Vielen Dank für Ihre Teilnahme, übermitteln Sie jetzt Ihre Antworten mit einem Klick auf Submit. | descriptive |
| 116 | [survey_schweiz_complete] | Section Header: *Form Status*<br>Complete? | dropdown<br>0 Incomplete<br>1 Unverified<br>2 Complete |
